# Fingers for signaling? A possible role of stromules in intracellular communication

**DOI:** 10.1093/plphys/kiag373

**Published:** 2026-06-16

**Authors:** Toranj Rahpeyma, Javier García Varo, Fabio Mühlberg, Peter Nick

**Affiliations:** Molecular Cell Biology, Joseph Kölreuter Institute of Plant Sciences, Karlsruhe Institute of Technology, Karlsruhe Germany; Molecular Cell Biology, Joseph Kölreuter Institute of Plant Sciences, Karlsruhe Institute of Technology, Karlsruhe Germany; Molecular Cell Biology, Joseph Kölreuter Institute of Plant Sciences, Karlsruhe Institute of Technology, Karlsruhe Germany; Molecular Cell Biology, Joseph Kölreuter Institute of Plant Sciences, Karlsruhe Institute of Technology, Karlsruhe Germany

## Abstract

Plastid stromules are frequently observed under stress, yet their function remains enigmatic. In the current study, we tested 2 alternative hypotheses: stromules might channel metabolic flux from plastids to peroxisomes during jasmonate biosynthesis, or they might serve as conduits for plastid–nucleus retrograde signaling. We used *Nicotiana tabacum* BY-2 cells to investigate stromulation in nonphotosynthetic plastids. Fluorescent markers for stroma, plastid outer membrane, peroxisomes, and the 12-oxo-phytodienoic acid (OPDA) exporter were combined with confocal visualization and AI-based quantification of stromule frequency and length. Exogenous methyl jasmonate (MeJA) and salicylic acid (SA) each triggered a rapid (∼60 min) ∼3-fold increase in stromule frequency. This increase primarily resulted from more initiation events, rather than elongation. Disrupting microtubules with oryzalin stopped MeJA from causing stromule formation, while actin depolymerization had no effect. Inhibition of jasmonate biosynthesis with Phenidone prevented SA-triggered stromule formation. MeJA-induced expression of genes involved in jasmonate biosynthesis (*AOC*) and jasmonate responses (*JAZ1* and *JAZ3*) was further amplified when stromule formation was suppressed by oryzalin, suggesting that stromules modulate jasmonate-dependent gene expression. The GFP-fusion of the OPDA exporter JASSY showed a significant preference for stromules, and over 80% of them associated with peroxisomes. Stromule frequency was highest during the cell-expansion phase. We discuss our findings in the context of a role for stromules in stress-dependent retrograde signaling from plastids to the nucleus.

## Teaser

Dynamic plastid extensions help plant cells modulate stress signaling and intracellular communication.

## Introduction

Stromules are stress-inducible plastid extensions, but their functional contribution to stress signaling remains unclear. In jasmonate biosynthesis, the plastid-derived intermediate 12-oxo-phytodienoic acid (OPDA) must be transferred to peroxisomes, and the efficiency of this step is likely influenced by plastid–peroxisome proximity. We therefore considered 2 scenarios. Stromules might facilitate OPDA transfer and thereby affect jasmonate biosynthesis (metabolic-flow scenario). Alternatively, stromules might contribute to plastid-to-nucleus retrograde signaling that modulates jasmonate-responsive gene expression (retrograde-signaling scenario). We addressed these questions in tobacco BY-2 cells by combining quantitative live-cell imaging with transcript-based readouts. Before presenting this approach, we briefly summarize current concepts of stromule identity, formation, and proposed functions.

Plastids can form tubular outgrowths filled with stroma, known as stromules. While described as early as the late 19th century (Haberlandt 1888; Senn 1908), they had fallen into oblivion. In a pioneering paper, they were rediscovered upon expression of plastid-targeted GFP in tobacco and *Petunia* (Kohler et al. 1997). In the same work, two-photon laser–mediated bleaching of a plastid reaching out to a neighboring plastid with a stromule showed recovery suggesting that GFP from the neighboring plastid replenished the bleached fluorophore leading to the conclusion that stromules connect plastids. This conclusion attracted considerable interest, because it challenged the notion of plastids as individual separate entities. However, soon after, the same lab published data demonstrating that photobleaching of seemingly interconnected plastids remains localized (Köhler and Hanson 2000). The concept of connected plastids was further accentuated by labeling the stroma with the photoactivatable monomeric (m)EOS. Here, the color change allows for higher sensitivity as compared to mere recovery after photobleaching (Schattat et al. 2012; Mathur et al. 2010). Again, plastids appeared as separate entities. This result was questioned in a rebuttal (Hanson and Sattarzadeh 2013) with the argument that the strong laser power in that experiment might have disrupted the connection, and using a weaker laser for photoconversion, examples for exchange of photoconverted mEOS from the irradiated to the non-irradiated plastid were presented. The reason for this discrepancy seems to be that incompletely divided plastids can be easily mistaken for plastids that entertain a stromule connection (for review, see [Schattat et al. 2015]). While the existence of stromules has been confirmed for numerous plants and under numerous conditions, their alleged function as bridges between neighboring plastids is meanwhile not even sustained by the lab that originally had created this idea (for review see [Hanson and Sattarzadeh 2013]).

The presence of stromules depends on tissue, developmental stage, and environmental conditions (Natesan et al. 2005). They are frequently observed when cells are exposed to biotic and abiotic stress. In contrast to the interconnection between incompletely divided plastids, true stromules are usually very thin (less than 1 µm), but long (up to 200 μm in tomato mesocarp cells). The cellular mechanisms behind stromule formation are still far from understood (for review, see [Hanson and Hines 2018]). Principally, they might form by local increases in the extensibility of the plastid envelope in concert with a more negative osmotic potential of the stroma. However, they might as well be produced by external forces, for instance, through adhesion of the plastid envelope to the cytoskeleton. When components of either the inner or the outer translocon (Tic and Toc) of the plastid import machinery were overexpressed, protrusions appeared that resembled stromules (Breuers et al. 2012). Here, a specific difference was observed—upon overexpression of the Tic component, these protrusions were not filled with stroma but corresponded to the intermembrane space. In contrast, overexpression of the Toc component led to protrusions that were filled with stroma, such that these authors concluded that they had induced stromules. These observations would speak in favor of a model where local changes in membrane composition would lead to stromulation. However, to what extent these conclusions based on electron microscopy can be transferred to the physiological context of stromulation has been questioned (for a critical appraisal, see [Delfosse et al. 2016]). A second argument for internal driving factors derives from experiments, where stromulation could be induced in isolated plastids (Brunkard et al. 2015), albeit at low efficacy. This efficacy could be strongly increased when a cytosolic extract was added to the isolated protoplasts (Ho and Theg 2016). When this extract was fractionated for molecular weight, it was the fraction comprising proteins with a molecular weight above 100 kDa that was responsible for this stimulation. The dependency on external proteins indicates that factors intrinsic to the plastid itself are not sufficient to induce stromulation.

Among potential external factors driving stromulation, the cytoskeleton is certainly a prime candidate. In fact, the disruption of stromulation by cytochalasin D or latrunculin B in tobacco leucoplasts suggests actin filaments as crucial elements (Kwok and Hanson 2004). In contrast, amiprophos-methyl or oryzalin seemed to play only a minor role in this regard. The dependence on actin might be either caused by an impact of actin assembly at the barbed end or, alternatively, by myosin-driven generation of force. The partial inhibition of stromules by the myosin ATPase inhibitor 2,3-butanedione 2-monoxime (Gray et al. 2001) indicated the participation of myosin motors. Fusions of class-XI myosin tails with GFP localized to the chloroplast envelope (Natesan et al. 2009). However, this localisation was not very specific, because other organelles were decorated as well. Experiments with transient RNAi based on C-terminal domains of myosin XI were able to block stromulation, which was taken as evidence that stromulation was myosin dependent (Natesan et al. 2009; Sattarzadeh et al. 2009). Likewise, silencing of CHUP1, a protein required for plastid mobility, harboring an actin-binding domain (Oikawa et al. 2003), was reported to promote the ground level of stromulation (Caplan et al. 2015). In contrast, stromules induced in the context of effector-triggered immunity were shown to be linked with microtubules and to become elongated when microtubule nucleation was perturbed by silencing a component of the γ-tubulin ring complex (Kumar et al. 2018). In this context, actin played a minor role, mainly in stabilizing preformed stromules. Thus, it is clear that the cytoskeleton is involved in stromulation, but the role of microtubules versus actin filaments seems to depend on the respective functional context.

While stromules have been observed under quite diverse conditions, a common theme seems to be that they appear when the cell is shifted to a stress, such that the redox homeostasis of the chloroplast is challenged (for review see [Hanson and Hines 2018]). Stromules can form in response to viral inducers of a hypersensitive reaction or bacterial infections, as well as their downstream signals, hydrogen peroxide and salicylic acid (Caplan et al. 2015), but also osmotic and salinity stress and their mediator abscisic acid (Gray et al. 2012). The energy state of the cell might play a role as well, since vacuum infiltration of either sucrose or glucose promoted stromulation significantly, while fructose or mannitol failed to do so (Schattat and Klösgen 2011).

What might be the function of stromules then? A straightforward idea is that the increased surface of the chloroplast should improve the interaction with other organelles, such as peroxisomes. In fact, the interaction of plastids with peroxisomes is essential for photorespiration that occurs under conditions of limited supply with carbon dioxide (for review, see [Bauwe et al. 2010]). Under the same conditions, the unbalanced electron transport over the thylakoid generates reactive oxygen species, which often associates with stromulation. The idea that stromules might act as players of organelle interaction is also supported by live-cell observations, where vesicles are shed from the stromule tip (Gunning 2005) and, thus, might sustain the flow from the outer plastid membrane to other compartments. Along the line of this concept, these vesicles deriving from the stromule tip could be interpreted as a kind of “extracellular vesicles” of the plastidic endosymbiont. In fact, extracellular vesicles have been described meanwhile from marine prokaryotes (Biller et al. 2014). A third idea derives from observations that stromules can be attracted by the nucleus (Erickson et al. 2017) and, therefore, might be involved in retrograde signaling of the plastid to the nucleus. Reports from chloroplast transformation, where recombinant proteins ended up in the nucleus (Isemer et al. 2012), would even claim compartmental contiguity between stroma and karyoplasm.

Of particular interest is the case of jasmonate biosynthesis. Here, the interaction between plastids and peroxisomes is required to process a signal generated in stress response, which subsequently orchestrates the adaptive responses to cope with this stress (for a recent comprehensive review, see [Wasternack and Song 2017]). The biosynthesis of jasmonates is initiated in the plastid culminating in formation of OPDA, which is transferred to the peroxisome and then subjected to β-oxidations. The resulting jasmonic acid can then either be methylated and used as a volatile signal, or it is conjugated to isoleucine giving rise to the bioactive hormone, jasmonate-isoleucine (JA-Ile). This is recognized by a receptor complex harboring the jasmonate ZIM-domain (JAZ) proteins that are then committed for proteolytic breakdown (Chini et al. 2007). Since this complex is suppressing the activity of the transcriptional activator MYC2, the proteolytic breakdown evoked by JA-Ile will turn on MYC2-dependent genes. This set of genes includes the *JAZ* genes themselves, such that the repressor is restored. This negative feedback loop reinforces a transient signature of signaling and safeguards the cell against constitutive overactivation of stress pathways (Wasternack and Song 2017).

Importantly, jasmonate biosynthesis requires metabolite transfer from the plastid to the peroxisome, which is dependent on membrane transporters. Export through the outer membrane of the plastid is brought about by the JASSY transporter (Guan et al. 2019), and import into the peroxisome by the ABC transporter COMATOSE (Theodoulou et al. 2005). Since the efficiency of this transfer is a bottleneck for the synthesis of jasmonate, it is most likely dependent on the degree of proximity between plastid and peroxisomal membranes. Thus, the formation of stromules might either facilitate or restrain the transfer of OPDA from the plastid to the peroxisome. We therefore asked whether stromules modulate OPDA transfer at the plastid–peroxisome interface and thereby affect jasmonate biosynthesis. To examine this question, we included salicylic acid (SA) alongside methyl jasmonate (MeJA). Whereas MeJA provides a downstream jasmonate signal, SA was used here as an upstream defense-related trigger. Given that SA also induced stromule formation, we used the LOX inhibitor phenidone to determine whether this response requires endogenous jasmonate biosynthesis, whereas exogenous MeJA was expected to bypass this biosynthetic step. Together, these comparisons indicate that stromule formation is associated with jasmonate through endogenous biosynthesis and/or downstream signaling, consistent with a central role of jasmonate in this response.

## Results

### Jasmonic and salicylic acid induce stromulation in tobacco BY-2

**Figure 1:**
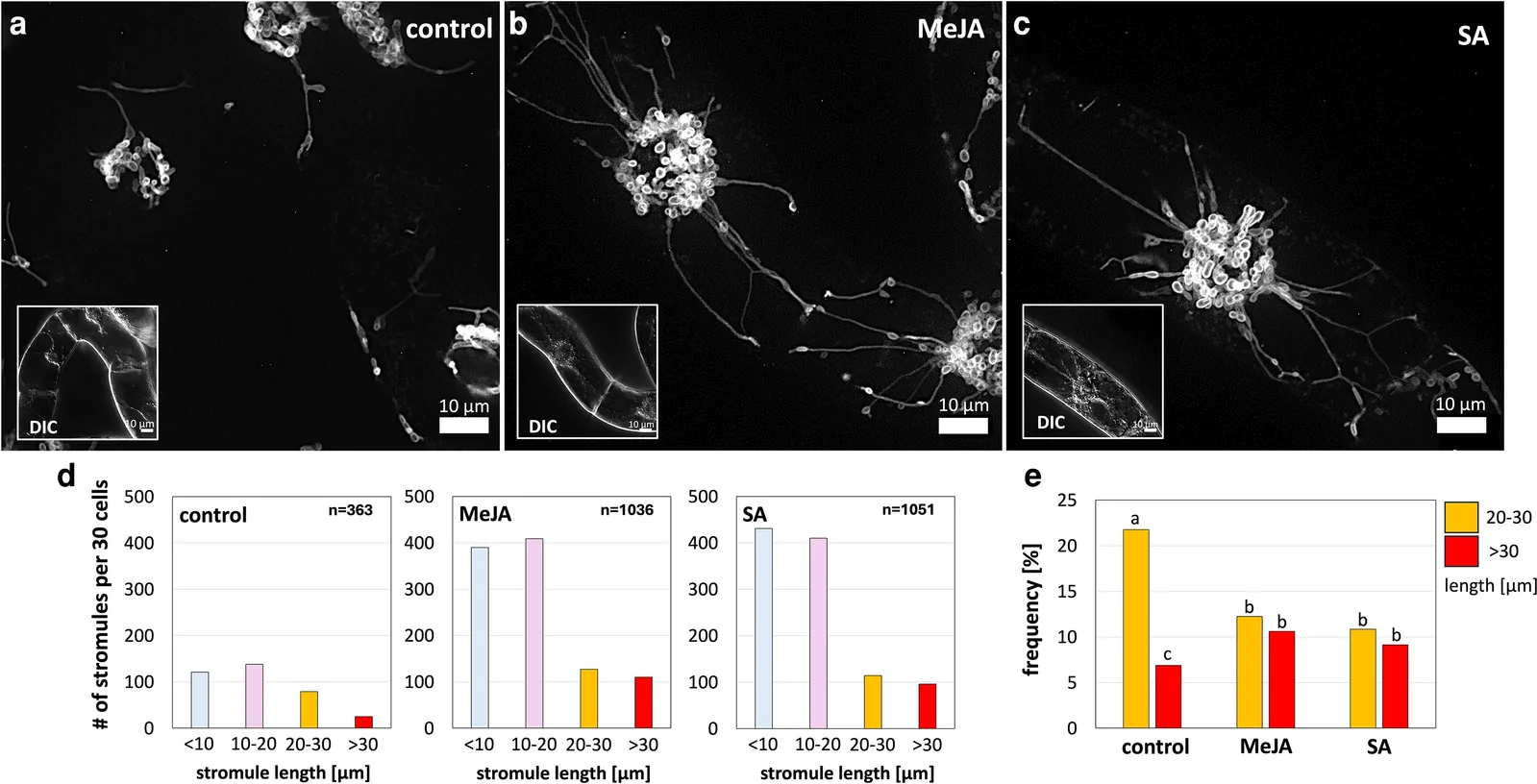
Induction of stromulation by stress-related phytohormones in tobacco BY-2 cells. a–c) Geometric projections of confocal z-stacks for representative cells expressing SENSITIVE TO FREEZING2 (SFR2) localized in the outer plastid membrane in fusion with mRFP. a) Untreated control cell. b) Cell after treatment with 100 µM of methyl jasmonate (MeJA) for 1 h. c) Cell after treatment with 100 µM of salicylic acid (SA) for 1 h. Each fluorescence panel includes a differential interference contrast image of the same cell to connect the topology of the stromule signal. d) Frequency distributions of stromule length for the 3 conditions. Very short stromules (<5 µm) were excluded. The bar chart corresponds to the incidence of stromules over a population of 30 cells. e) Shifts in frequency for stromules 20 to 30 µm and >30 µm in length. Different letters indicate differences significant at *P* < 0.05 based on the nonparametric Fligner-Killeen test.

Since stromules have often been observed in the context of stress, we tested whether they can be induced by the stress hormones MeJA or SA. Since both hormones can act antagonistically or in synergy, depending on the stress context, we tested them independently. The outer membrane of the plastid was visualized by the marker SENSITIVE TO FREEZING2 (SFR2) in fusion with red fluorescent protein (RFP) (Mathur et al. 2022). Under control conditions, only very few and very short protrusions emerged from the plastid (Fig. 1a). However, 60 min after treatment with 100 µM of either MeJA (Fig. 1b) or SA (Fig. 1c), these protrusions became numerous and long. To test whether these protrusions were stromules, we repeated the experiments with cells expressing the stroma marker tpFNR-mEOS (Schattat et al. 2012) and observed the same phenomenon, ie a strong induction of long stromules by both MeJA (Figure S1B) and SA (Figure S1C), which was not seen under control conditions (Figure S1A). To see whether stromulation can be induced also by endogenous jasmonates, we subjected the cells to moderate salt stress (100 mM NaCl, 1 h), which can induce the bioactive isoleucine conjugate, JA-Ile (Asfaw et al. 2022). Salt stress induced a clear stromulation as well (Figure S1D). To quantify the responses, we developed an AI-driven methodology to automatically recognize and measure stromules visualized by the SFR2 marker. This quantification revealed a skewed length distribution with a preponderance of short stromules (Figure S2). The difference between the treatments concerned mainly the overall incidence of stromules (Fig. 1d), which was around 3 times lower under control conditions as compared to the treatments with MeJA or SA. If these differences over global incidence were ignored, it was mainly the frequency of very long stromules (>30 µm), where differences could be observed (Fig. 1e). Such very long stromules were significantly more common after treatment with MeJA and SA, while stromules that were between 20 and 30 µm became less frequent. However, it should be noted that, overall, only around 30% of stromules reached a length of 20 µm or more; the majority was shorter. As a summary, we observed that MeJA and SA induced stromulation rapidly. The induction was mainly due to a higher number of stromulation events, and less to stimulation of stromule length. The many fewer (only a third) stromules seen under control conditions had a similar chance (around 30%) to elongate to 20 µm or more. In addition, the 2 hormonal treatments showed a mild stimulation of elongation into very long structures that could span 30 µm or more.

### SA-dependent stromulation requires jasmonate synthesis

**Figure 2:**
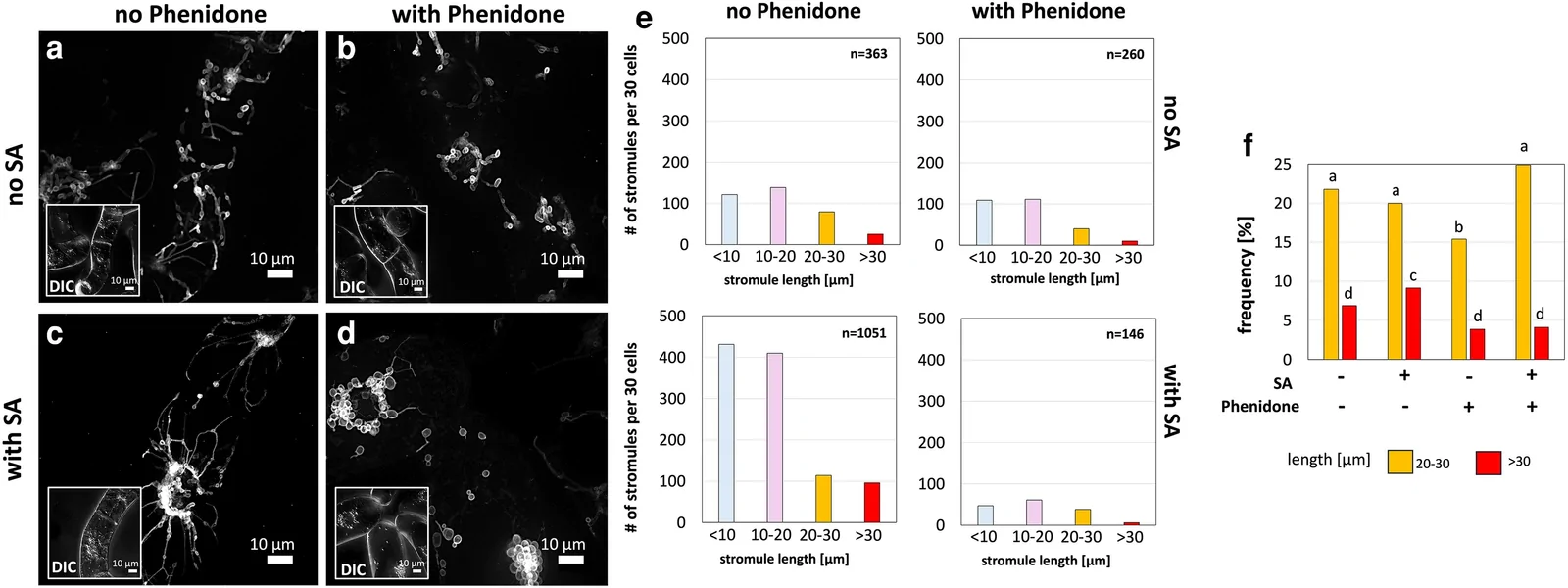
Salicylic acid (SA)-induced stromulation can be blocked by phenidone, a lipoxygenase inhibitor. Representative tobacco BY-2 cells expressing SENSITIVE TO FREEZING2 (SFR2) localized in the outer plastid membrane in fusion with mRFP are shown. Images are geometric projections of confocal z-stacks. a) Untreated control. b) Treatment with 2 mM of phenidone for 90 min. c) Treatment with 100 µM of SA for 60 min. d) Treatment with 100 µM of SA following a pretreatment with 2 mM phenidone for 30 min. A differential interference contrast imaging of the same cell within each fluorescence panel shows a topological comparison of stromule signal. e) Frequency distributions of stromule length for the 4 conditions. f) Shifts in frequency for stromules 20 to 30 µm and >30 µm in length. Different letters indicate differences significant at *P* < 0.05 based on the nonparametric Fligner-Killeen test.

The 2 hormones, MeJA and SA, might induce stromules independently. Alternatively, the effect of SA might be indirect through synthesis of endogenous jasmonates. To distinguish these options, we probed stromulation in response to SA after pretreatment with phenidone, a lipoxygenase inhibitor (Bruinsma et al. 2010), thereby interfering with the initial step of oxylipin biosynthesis. In the absence of phenidone, SA induced a rapid and efficient formation of stromules (Fig. 2a, c), while a pretreatment with phenidone (2 mM, 30 min) thoroughly inhibited the stromulation response to SA (Fig. 2b, d). Again, the inhibition was mainly acting on the incidence of stromulation events (Fig. 2e). Here, SA stimulated by around 3-fold, while phenidone suppressed incidence to around half of the resting level. In contrast, stromule elongation (Fig. 2f) was only mildly changed by phenidone. Thus, SA requires jasmonate synthesis to induce stromulation; this induction acts mainly on the initiation of stromules, much less so on the elongation of already initiated stromules.

### Stromulation requires microtubules, while actin filaments are dispensable

**Figure 3:**
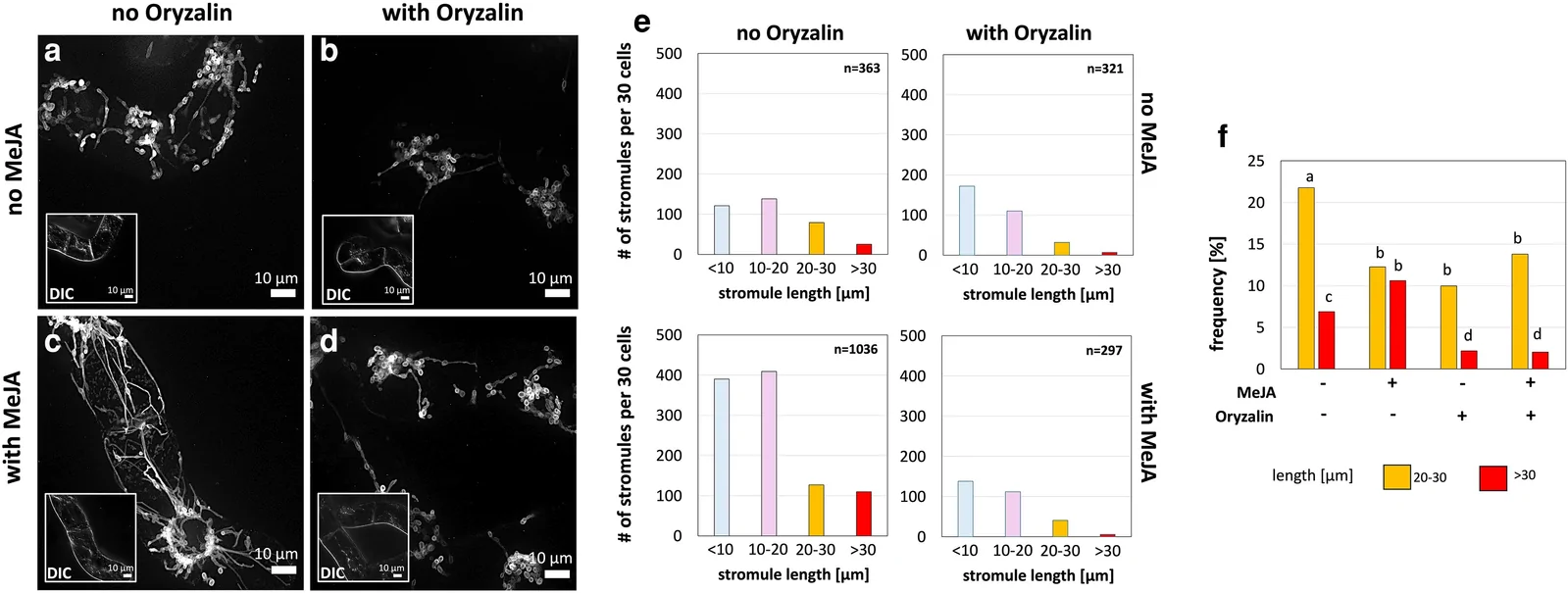
MeJA-induced stromulation can be blocked by oryzalin, a microtubule-eliminating herbicide. Representative tobacco BY-2 cells expressing SENSITIVE TO FREEZING2 (SFR2) localized in the outer plastid membrane in fusion with mRFP are shown. Images are geometric projections of confocal z-stacks. a) Untreated control. b) Treatment with 100 µM of oryzalin for 120 min. c) Treatment with 100 µM of MeJA for 60 min. d) Treatment with 100 µM of MeJA following a pretreatment with 10 µM of oryzalin for 60 min. A differential interference contrast image of the same cell is embedded in each fluorescence panel to provide topological context for the stromule signal. e) Frequency distributions of stromule length for the 4 conditions. f) Shifts in frequency for stromules 20 to 30 µm and >30 µm in length. Different letters indicate differences significant at *P* < 0.05 based on the nonparametric Fligner-Killeen test.

To get insight into the cellular mechanisms of stromulation, we probed stromulation by MeJA after pretreatment with 100 µM oryzalin for 2 hours, a treatment that fully eliminates microtubules. While MeJA (100 µM, 60 min) induced numerous stromules (Fig. 3c), the oryzalin pretreatment completely abolished this response (Fig. 3d), accompanied with a significant depletion of very long stromules (Figs. 3e, f). Oryzalin suppressed also the endogenous stromulation in absence of MeJA (Fig. 3b versus Fig. 3a). This was reflected in a low incidence of stromules longer than 30 µm (Fig. 3e, f). To check the specificity of suppression, we also probed the impact of actin filaments using a pretreatment with latrunculin B (Figure S3). Here, the stromulation induced by MeJA was able to proceed unimpaired. Thus, while intact microtubules were found to be necessary for stromulation in response to MeJA, actin filaments seemed to be dispensable (Figure S4).

### Stromules respond by incidence, rather than by length

**Figure 4:**
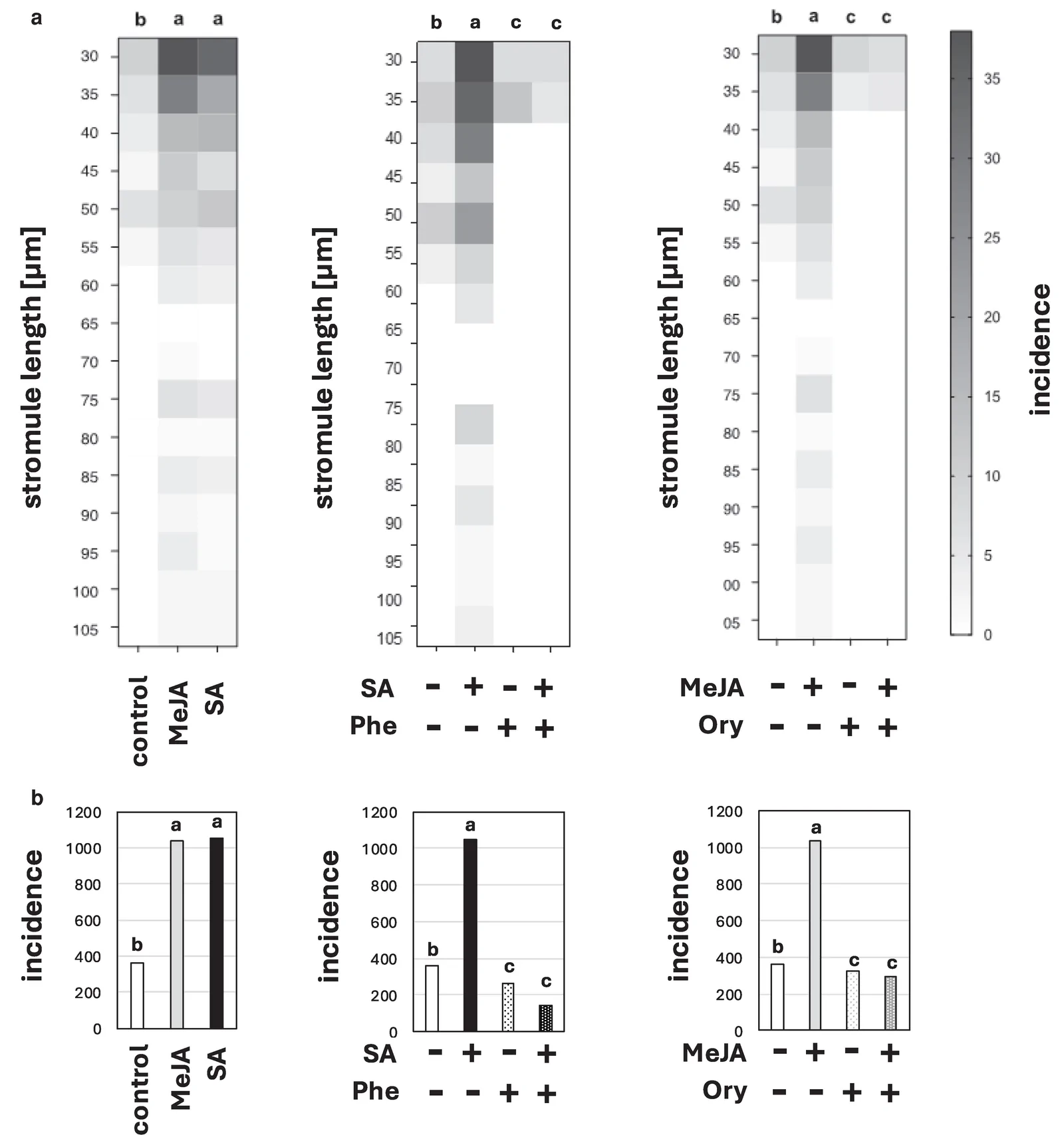
Incidence of stromules depending on their length and treatment. a) Distribution over different length classes (numbers indicate the center of the respective class). b) Total incidence of stromules. Incidence refers to the total number of stromules detected by the AI-driven image analysis algorithm in a population of 30 individual cells. Conditions are control (untreated); MeJA, 100 µM of methyl jasmonate for 60 min; SA, 100 µM of salicylic acid for 60 min; Phe, 2 mM of phenidone for 90 min; Phe + SA, 100 µM of SA following a pretreatment with 2 mM phenidone for 30 min; Ory, 100 µM of oryzalin for 120 min; Ory + MeJA, 100 µM of MeJA following a pretreatment with 10 µM of oryzalin for 60 min. Different letters in panels a and b indicate differences significant at *P* < 0.05 based on the nonparametric Fligner-Killeen test.

Our AI-based stromule detection enabled quantification of number and length in a large number of cells. All frequency distributions over stromule length were left skewed (Figure S2), meaning that short stromules were overrepresented. To detect potential differences in the incidence of very long stromules (that were masked by the strong skew of the distribution), a cutoff at <30 µm was applied (Fig. 4a). This revealed that, in fact, MeJA and SA increased the prevalence of very long stromules as compared to the control or as compared to treatment with phenidone or oryzalin. However, only a minority of stromules reached lengths of 30 µm or more (Figure S2). Differences in stromule incidence were much more salient (Fig. 4b). Here, both MeJA and SA induced the frequency of stromulation events by more than 2.5-fold. Likewise, phenidone suppressed SA-induced stromulation even beyond the resting level seen in untreated controls, and the same holds true for the suppression of the stromulation response to MeJA after pretreatment with oryzalin.

### Stromule formation modulates the cellular regulation of specific genes in JA synthesis and response

**Figure 5:**
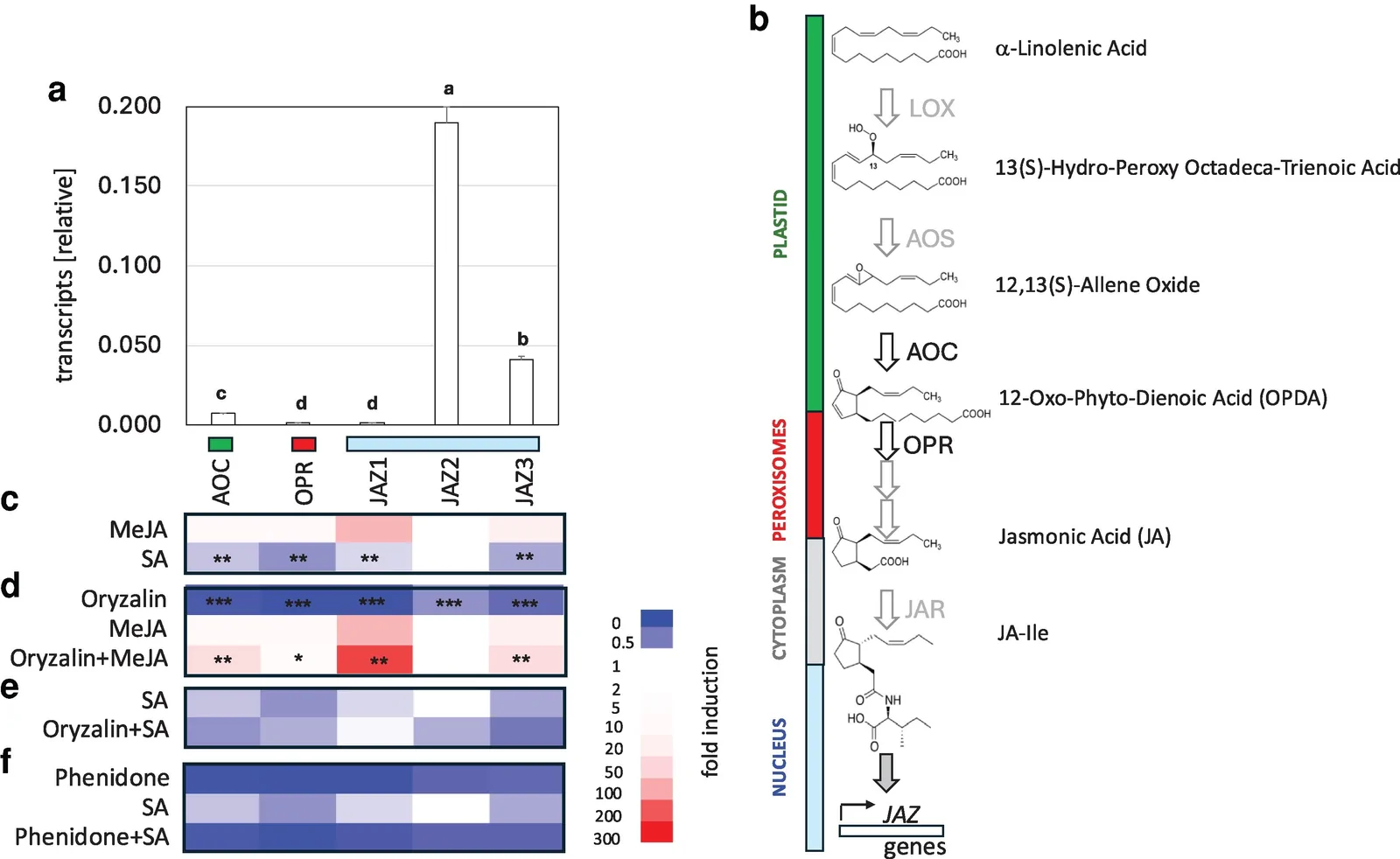
Response of jasmonate synthesis and jasmonate response genes to factors that also modulate stromulation. a) Steady-state transcript levels for allene oxide cyclase (*AOC*), OPDA reductase (*OPR*), and jasmonate response genes *JAZ1*, *JAZ2*, *JAZ3* in relative units. b) Intracellular compartmentalization of jasmonate biosynthesis and response to show the localization of the gene products for the tested transcripts. c) Response of gene expression to MeJA and SA. d) Dependence of the MeJA response on oryzalin, eliminating microtubules. e) Dependence of the SA response on oryzalin. f) Dependence of the SA response on phenidone. Conditions are: MeJA, 100 µM of methyl jasmonate for 60 min; SA, 100 µM of salicylic acid for 60 min; Oryzalin, 10 µM of oryzalin for 120 min; Oryzalin + MeJA, 100 µM of MeJA for 60 min following a pretreatment with 10 µM of oryzalin for 60 min; Oryzalin + SA 100 µM of SA for 60 min following a pretreatment with 10 µM of oryzalin for 60 min. Data represent means of 3 independent experimental series, each in technical triplicates. c–f) Fold-induction relative to the untreated control values. Statistical significance was analyzed by one-way ANOVA followed by Duncan's multiple range test. Asterisks in heat maps indicate significant differences versus the hormone-only treatment (MeJA in c and d, SA in e and f), whereas in a, different letters indicate significant differences among groups. Fold-change values were calculated relative to the untreated control; values below 1 therefore indicate suppression of the respective gene (*P < 0.05, **P < 0.01, ***P < 0.001).

The biosynthesis and signaling of jasmonates involve several cellular compartments (Fig. 5b). The first committed step of the jasmonate pathway is the oxidation of α-linolenic acid in the plastid leading to OPDA, which is exported from the plastid and imported into the peroxisome, where a series of beta-oxidations generates jasmonic acid that exits the peroxisome and is conjugated in the cytoplasm with isoleucine to yield the active form, JA-Ile. Binding to the receptor complex resulting in the proteolytic degradation of the JAZ proteins is an event happening in the nucleus. Since activation of *JAZ* genes is among the earliest responses, their induction can be used as proxy for the presence of JA-Ile. We asked whether the transcript levels for these transcripts would depend on stromulation. To address this experimentally, we searched for a condition where *JAZ* genes are activated in the absence of stromules. Since stromules require microtubules for their formation, we used oryzalin, a compound that specifically and efficiently eliminates plant microtubules. In addition to the *JAZ* genes, we also included, for comparison, transcripts for allene oxide cyclase (*AOC*), as last plastidic, and OPDA reductase, as first peroxisomal step of JA synthesis. With the exception of *JAZ2*, the resting levels of all transcripts were low (Fig. 5a), but became stimulated after 60 min of treatment with MeJA, while SA resulted mostly in fold-changes below 1 (Fig. 5c). While pretreatment with oryzalin did not change the MeJA responses of *OPR*, or the abundant *JAZ2*, the induction of *AOC*, *JAZ3*, and, most saliently, of *JAZ1* was clearly amplified (Fig. 5d), although oryzalin, by itself, was not inducing, but rather suppressing the transcripts of these genes. With SA as trigger, transcript levels were generally reduced, and oryzalin pretreatment did not significantly modify this response; in all cases, fold changes remained below the untreated control (fold change < 1) (Fig. 5e).

A pretreatment with phenidone prior to application of SA suppressed gene expression to levels below the untreated control (Fig. 5f). Thus, the markedly enhanced transcriptional response observed under stromule-deficient conditions is consistent with stromules acting as negative modulators for the MeJA-triggered induction of specific transcripts for the plastidic part of jasmonate biosynthesis (*AOC*) and jasmonate response (*JAZ1, 3*).

### The regulation of OPR transcripts differs from genes in early JA synthesis and JA response

**Figure 6:**
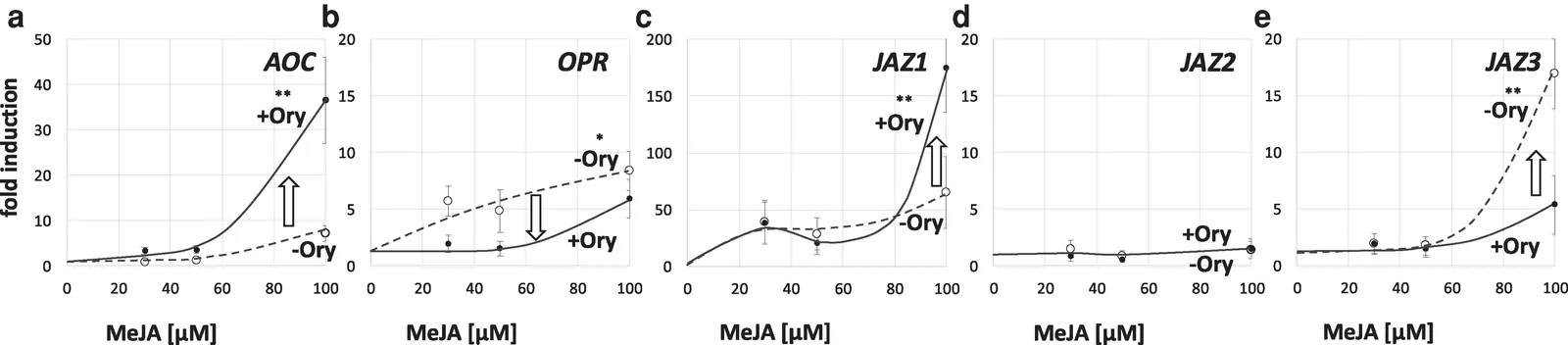
Dose–response of jasmonate synthesis and jasmonate response genes over exogenous jasmonic acid either without (–Ory) or with (+Ory) pretreatment with 10 µM of oryzalin prior to addition of jasmonic acid and incubation for an additional 60 min. Induction of steady-state transcript levels for allene oxide cyclase (*AOC*, a), OPDA reductase (*OPR*, b), and jasmonate response genes *JAZ1* (c), *JAZ2* (d), or *JAZ3* (e) over untreated control cells are shown. Data represent means ± SE of 3 independent experimental series, each in technical triplicates. Solid lines and filled circles indicate +Ory, whereas dashed lines and open circles indicate −Ory. Arrows indicate the effect of oryzalin treatment. The arrows demonstrate the effect of the oryzalin treatment. Note the 10-fold compression of the *y* axis in c. Asterisks indicate statistically significant differences as determined by one-way ANOVA (*P < 0.05, **P < 0.01).

To test whether the effect of stromules on transcript accumulation is a general phenomenon or whether it depends on the type of transcript, we probed the dose–response using different concentrations of MeJA either in presence or absence of oryzalin pretreatment. Here, clear qualitative and quantitative differences were observed (Fig. 6). Oryzalin stimulated the induction of *AOC* (Fig. 6a), *JAZ1* (Fig. 6c), and *JAZ3* (Fig. 6e) by more than 3-fold, but only for high concentrations of MeJA exceeding 50 µM. Instead, the highly expressed *JAZ2* was not responsive, neither to MeJA nor oryzalin (Fig. 6d). The response of *OPR* differed concerning quality and sensitivity (Fig. 6b). In the absence of oryzalin, *OPR* was already induced by 30 µM MeJA and increased only slightly, when the concentration was raised to 100 µM. Pretreatment with oryzalin shifted this dose–response curve to the right, approximately by a factor of 3, meaning that the otherwise sensitive MeJA of this gene was not amplified as for the other genes, but suppressed. Thus, the regulatory impact of stromulation differs for transcripts of proteins acting in the plastid (*AOC*) or the nucleus (*JAZ1*, *JAZ3*) versus *OPR*, acting in the peroxisome.

### The OPDA exporter JASSY is partitioned to stromules

**Figure 7:**
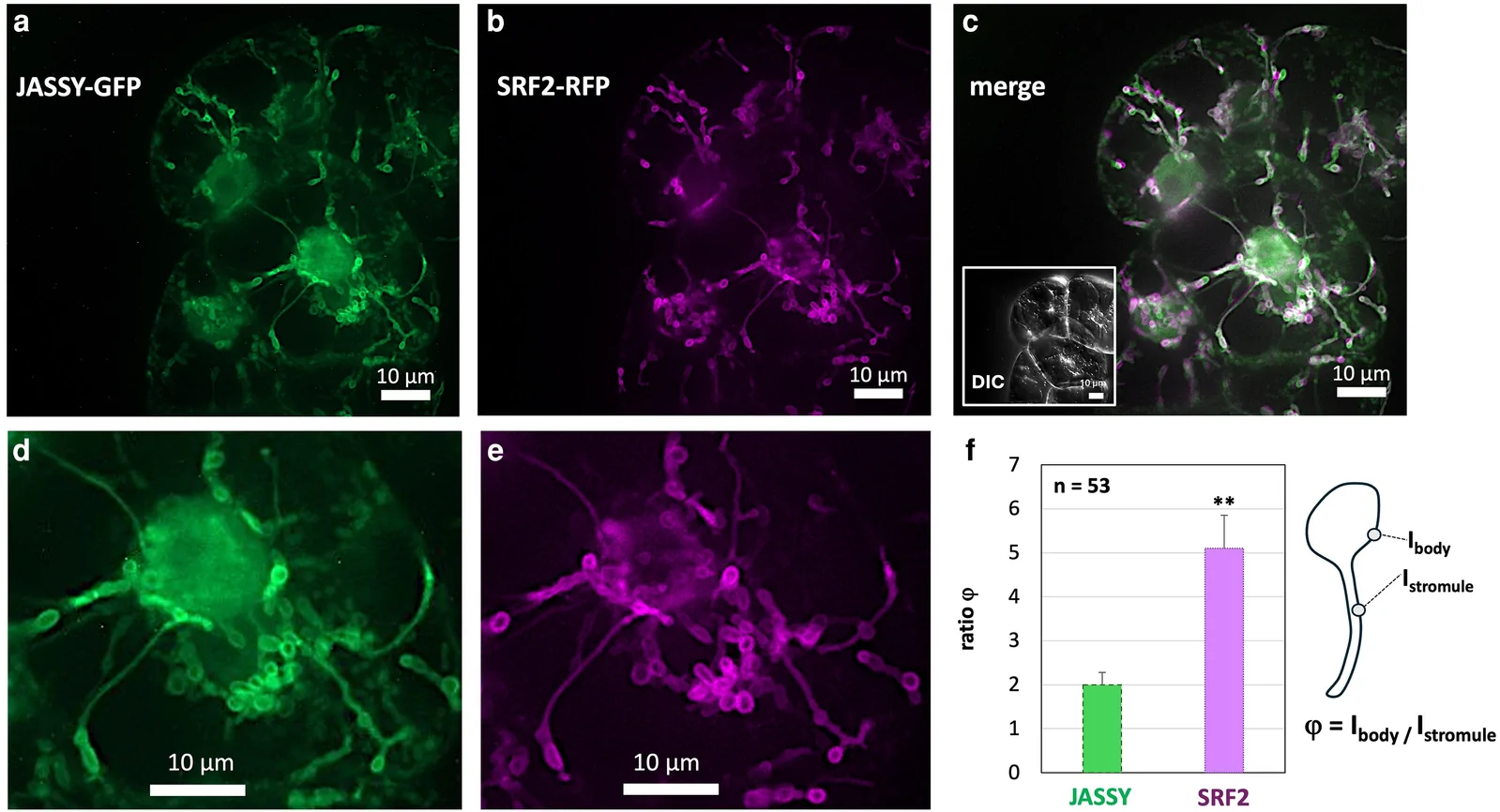
Subcellular distribution of the OPDA exporter JASSY relative to the plastid outer membrane. a, d) JASSY-GFP signal in the overview and magnified perinuclear region, respectively. b, e) Corresponding signal of the outer membrane marker SFR2-RFP. c) Overlay of both signals; the differential interference contrast in the merged panel reveals the cell structure. Images are geometric projections of confocal z-stacks. f) Quantification of signal heterogeneity between the plastid body and stromule. The bar graph shows the ratio *φ* = *I_body_/I_stromule_*, where *I_body_* represents the fluorescence intensity of the plastid body (JASSY), and *I_stromule_* represents the fluorescence intensity of the stromule (SFR2). Data are presented as means ± SE (n = 53). The difference in the ratio was significant at *P* < 0.01 based on a χ^2^ test.

We wondered whether stromules would interfere with jasmonate biosynthesis. Here, a crucial step is the export of OPDA from the plastid, enabling further processing in the peroxisome (Fig. 5b). This step is mediated by the exporter JASSY (Guan et al. 2019). Therefore, we followed the subcellular localization of JASSY-GFP in the background of the SFR2-RFP line. As expected, the JASSY signal (Fig. 7a) was seen in the outer membrane (Fig. 7b). The overlap with SFR2-RFP was only partial, though. In the merged image (Fig. 7c), the yellow signals reporting co-localization were accompanied by red and green regions, indicating preponderance of either SFR2 or of JASSY. Here, the labelling of stromules with JASSY was more pronounced as compared to that with SFR2 (Fig. 7d, e). To test this impression, the ratio of signal in the plastid body over the signal in the stromule was quantified on a mean grey value base (Fig. 7f). For SFR2, this ratio was ∼5, meaning that this marker was strongly enriched in the plastid body. For JASSY, this ratio was only ∼2, meaning that, here, a significantly larger proportion of this transporter was localized to stromules. Thus, compared to SFR2 as general marker for the outer plastid membrane, JASSY was partitioned to stromules. The JASSY-GFP signal was nonuniform along the plastid envelope and appeared enriched on stromules compared with the general outer-envelope marker SFR2 (Fig. 7d–f). Our NND results extend this finding by demonstrating that peroxisomes are nonrandomly positioned in close proximity to stromules. Such spatial alignment likely creates a more direct OPDA transfer route from plastids to peroxisomes.

### Most stromules are decorated with peroxisomes

**Figure 8:**
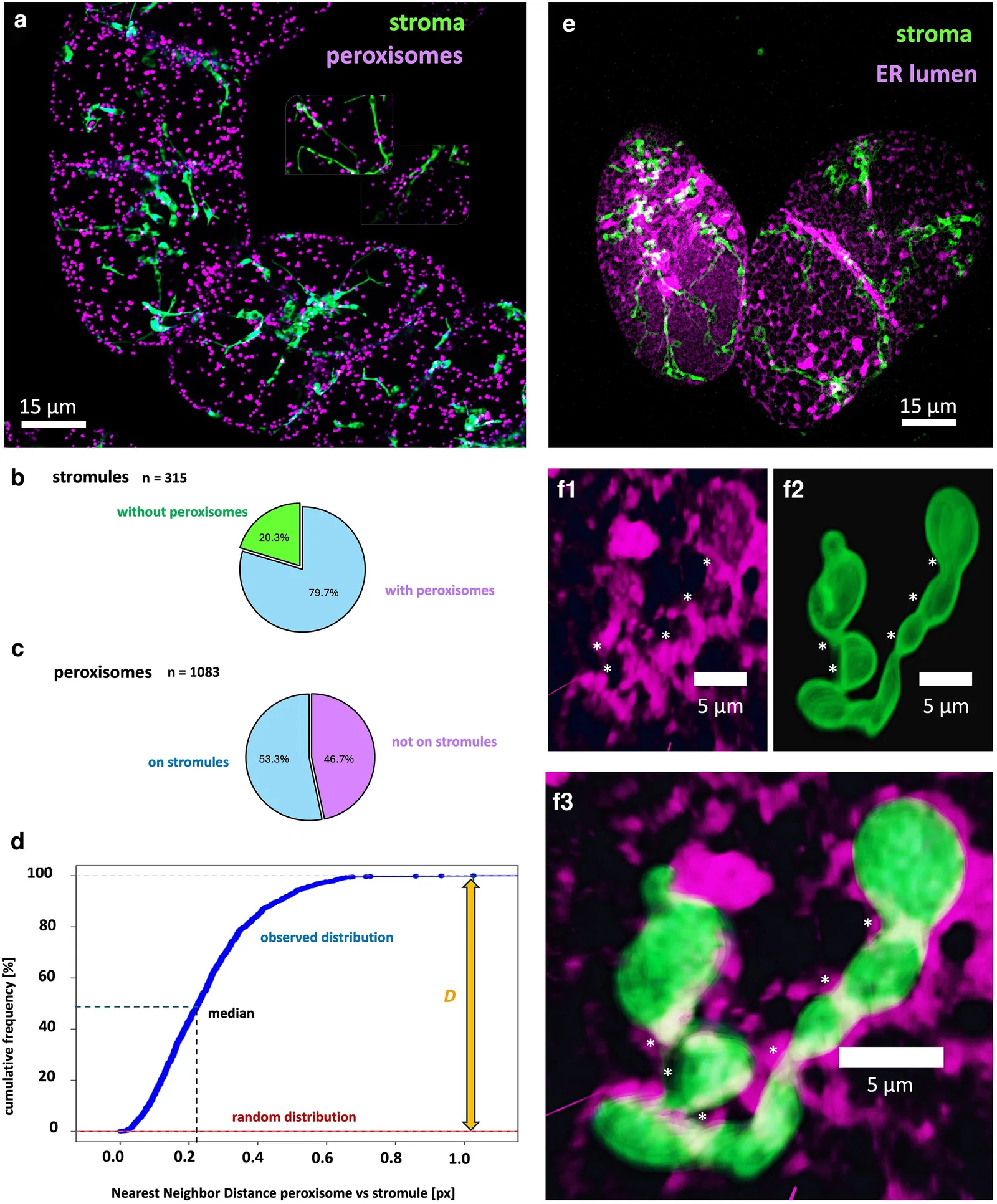
Relationship between stromules and other organelles as assessed by double visualization with the stroma marker N-terminal fragment of ferredoxin NADPH oxidoreductase (tpFNR-mEOS, green). a–d) Relation between stromules and peroxisomes (visualized by the marker PTS1-mCherry). a) geometric projection of a confocal z-stack through the cells is shown to illustrate the total population of peroxisomes. b, c) Mutual colocalisation scored from individual confocal sections of a stack (to exclude false positive values from overlay of different z-planes). b) Frequency of stromules not adjacent to peroxisomes (green) compared with that of stromules adjacent to peroxisomes (blue). c) Frequency of peroxisomes not adjacent to a stromule (magenta) as compared to that of peroxisomes adjacent to a stromule (blue). d) Cumulative distribution function (CDF) of nearest neighbor distances (NNDs; shortest Euclidean distances) from each peroxisome to the closest stromule. The observed CDF (blue) is compared with a randomized control generated by repositioning the same number of peroxisomes within the same image boundaries (red). The observed distribution is shifted toward shorter distances (median ≈ 0.2 pixels) relative to the randomized control (2-sample Kolmogorov–Smirnov test: D = 1, *P* < 0.0001). The orange arrow indicates D, the maximum difference between the observed and randomized distributions, consistent with a nonrandom spatial association (enrichment) of peroxisomes near stromules. Stromulation was induced by treatment with 100 µM MeJA for 60 min. e–f) Relation between stromules and the ER lumen (visualized by the marker HDEL-RFP). e) Geometric projection of a z-stack collected over a representative cell, showing plastid bodies and stromules closely adjacent to the ER tubules and extending through the ER polygons. f) 3D-rendered views highlighting this close association; F1 shows the ER lumen signal, F2 the stromal signal, and F3 the merged rendering. White asterisks indicate stromule constrictions at sites of overlap with the ER.

To test whether the partitioning of JASSY to stromules links with an interaction between stromules and peroxisomes, we generated a double transformant expressing tpFNR-mEOS alongside the peroxisomal marker PTS1-mCherry, enabling the visualization of stromules (induced by 100 µM MeJA for 60 min) in green and peroxisomes in red. In geometrical projections (Fig. 8a), numerous peroxisomes could be observed. Some of them were adjacent to stromules, others were not. Since in geometrical projections, it is difficult to distinguish whether an apparent association of 2 structures is just caused by seeing these structures at the same XY-position, while the Z-position might be quite different, we analyzed the individual confocal sections of a stack. Here, we scored 79.7% of stromules as being adjacent to at least one peroxisome (Fig. 8b). For peroxisomes, the association was less evident (Fig. 8c). Here, 53.3% of peroxisomes associated with stromules, while 46.7% showed no stromule association. To statistically validate the association for the subset of peroxisomes close to a stromule, we performed a nearest neighbor distance (NND) analysis (Fig. 8d). Herefore, we constructed the cumulative frequency distribution over the shortest distances from the peroxisome to the next stromule. The median was at 0.2 pixels. As median for a random distribution, a value of 400 pixels would be expected. The difference was tested by a nonparametric Kolmogorov-Smirnov test and found to be highly significant (*P* < 0.0001), indicating that peroxisomes are nonrandomly positioned in close proximity to stromules compared with a randomized reference distribution (Fig. 8d).

### Stromules are tightly connected to the ER

To further explore the spatial association of stromules with the endomembrane system, BY-2 cells stably expressing ER-lumen marker HDEL-RFP and tpFNR-mEOS were generated. Both plastid bodies and stromules were closely adjacent to the ER tubules and reached through the polygons (Fig. 8e). Details of this proximity were visualized through 3D rendering (Fig. 8F1–3). The stromules showed distinct constrictions at the sites where both structures overlap (Fig. 8F1–3, white asterisks).

### Endogenous stromulation is very active during cell expansion

**Figure 9:**
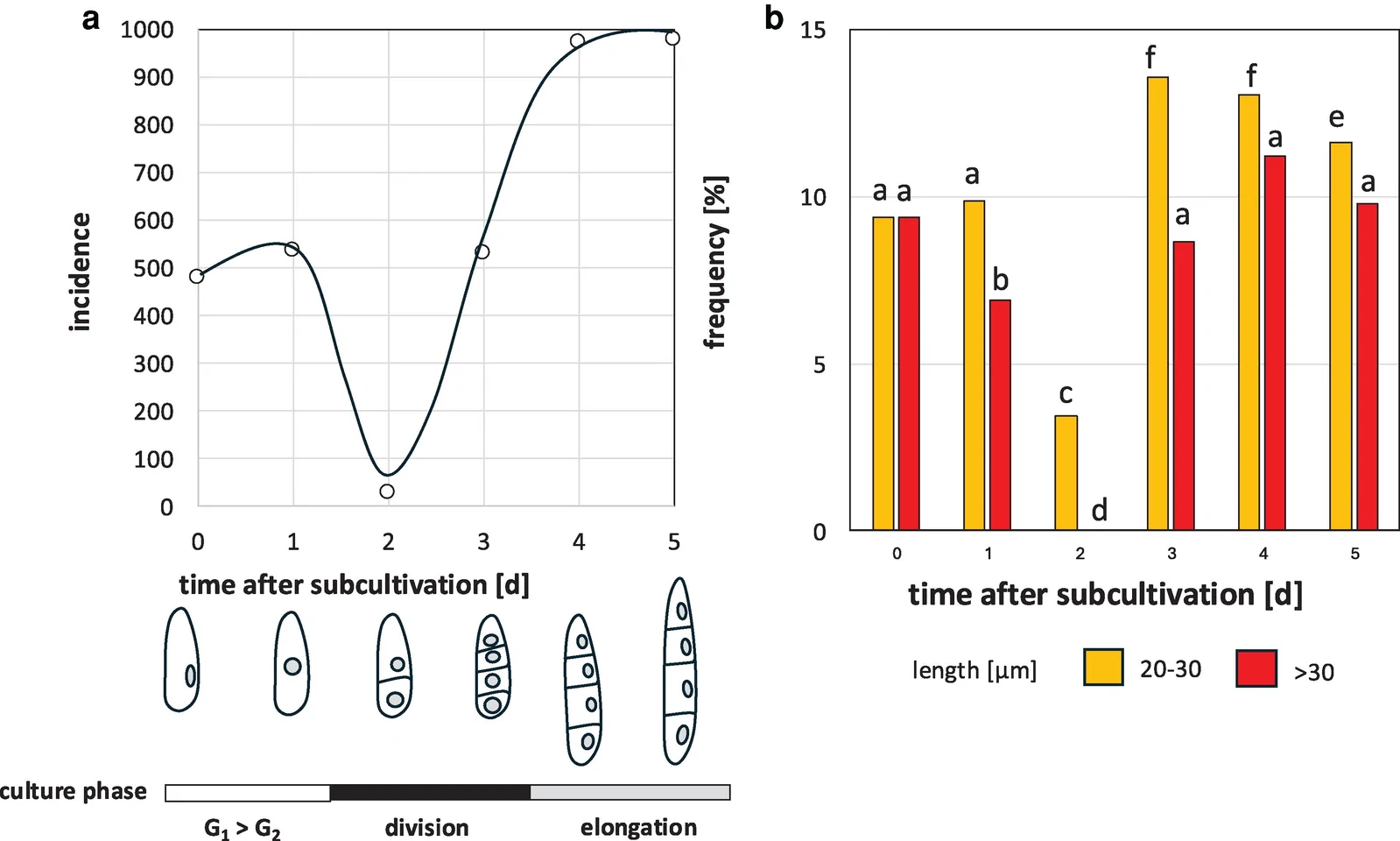
Dependence of endogenous stromulation on the phase of the cultivation cycle in tobacco BY-2 cells expressing SENSITIVE TO FREEZING2 (SFR2) localized in the outer plastid membrane in fusion with mRFP. Shifts in total number of stromules (a) and frequency of stromules 20 to 30 µm and >30 µm in length (b) are shown. Data points represent a population of 30 individual cells from 3 independent experimental series. Different letters indicate differences significant at *P* < 0.05 based on the nonparametric Fligner-Killeen test.

A well-kept suspension culture of tobacco BY-2 cells exhibits a distinct sequence of developmental events (Huang et al. 2017). Upon subcultivation, the cells need around 1 d to shift from G_1_ to G_2_ (Fig. 9a), followed by around 2 d of mitotic activity giving rise to files of mostly 4 cells easing off around day 3 to 4. Then, cells elongate over the following 2 d, and subsequently the files progressively decay into individual cells. We wanted to know whether the differences in cell state would be reflected in stromulation. As to avoid perturbance of the developmental responses, we refrained from triggering stromulation by hormones and just focused on endogenous stromulation. We observed that the incidence of stromules dropped sharply, by almost 5 times, after d 1, but had recovered to the initial value on d 3, continued to grow to twice of the initial value and stayed high also during the subsequent day (Fig. 9a). This pattern was reflected in the frequency of long stromules with respect to the sharp drop on d 2 (Fig. 9b), albeit the frequencies prior and subsequent to the division phase were comparable. Thus, stromulation is most active during cell expansion and clearly inhibited in cycling cells.

## Discussion

The current work addressed the cellular function of stromules in tobacco BY-2 cells, using fluorescent markers for either stroma or the outer plastidic membrane. These cells are void of chlorophyll, such that one deals with leucoplasts. While leucoplasts are prone to irregularities of shape, both kinetic and morphological criteria support the notion of genuine stromules: they appear swiftly within 30 min of MeJA or SA treatment, which by far exceeds the dynamics of plastid deformations, and they are consistently narrower than 1 µm, in contrast to plastid bodies, which exceed this width. Quantification based on AI-assisted automatic recognition showed that the inception of new stromules was far more responsive than the elongation of preexisting stromules. Stromulation required intact microtubules but was independent of actin filaments. Suppression of stromulation by oryzalin amplified the induction of specific members of jasmonate biosynthesis and response. We also found evidence for an interaction between stromules and peroxisomes.

These findings raise 2 questions. First, what is the role of microtubules in stromulation? Our data show that depolymerization of microtubules by oryzalin suppresses basal stromulation and abolishes MeJA-induced stromule formation, primarily by reducing stromule incidence (Figs. 3–4), whereas actin depolymerization does not impair stromulation (Figure S3–4). Second, do stromules modulate MeJA-dependent gene expression by channeling OPDA metabolism, or do they serve as retrograde signaling conduits between plastids and the nucleus? We consider these 2 models below in light of our findings.

### Jasmonate-induced stromules require interaction with microtubules

Oryzalin suppressed stromulation efficiently, demonstrating that microtubules are necessary (Fig. 3). The quantitative treatment (Fig. 3d, f, Fig. 4) revealed that the target of oryzalin was mainly the incidence of stromules, only to a minor extent their elongation. Similar studies in nongreen plastids of tobacco reported that both microtubules and actin can influence stromule morphology, but did not quantify incidence changes in detail (Kwok and Hanson 2003). Our BY-2 data extend this work by showing that, in leucoplasts, microtubules play a dominant role in stromule incidence, whereas actin filaments are not required under our conditions. In our hands, elimination of F-actin by latrunculin B did not impair stromulation (Figure S3), although a minority of actin filaments were found to align with stromules (Figure S4). Whether the difference in actin dependency is linked to the difference between etioplasts (hypocotyl) and leucoplasts (tobacco BY-2 cells) remains to be elucidated. Using transient transformation in *Nicotiana benthamiana* leaves, Kumar et al. (2018) reported that stromules extend along microtubules and alterations in microtubule organization, including chemical stabilization by Taxol or genetic perturbation of γTuRC components, are associated with increased stromule incidence and length in epidermal pavement cells (Kumar et al. 2018). These observations are consistent with our findings, because they represent different microtubule perturbations: in our system, microtubule depolymerization by oryzalin strongly suppresses MeJA-induced stromulation (Figs. 3–4), whereas microtubule stabilization/reorganization may promote stromule dynamics in other experimental contexts. Together, these results support a context-dependent role of microtubules in stromule formation and dynamics.

Stromule incidence is more strongly affected than stromule length, consistent with a 2-phase model where microtubules primarily affect stromule initiation: Oryzalin strongly reduces total stromule incidence (Fig. 4b, subpanel 3). Moreover, oryzalin reduced the frequency of very long stromules (Figs. 3e–f, 4a, subpanel 3), suggesting that microtubules may also contribute to the maintenance and/or elongation of long stromules. In tobacco pavement cells, a triple label of microtubules, endoplasmic reticulum (ER), and plastids showed that the ER wrapped both stromules and microtubules aligning with them, illustrating the general concept of the ER as a scaffold for organelle interaction (reviewed in [Mathur et al. 2022]). In the same system, a crucial role of a class-XIV kinesin, later termed as KIS1 (kinesin required for inducing stromules), could be established (Meier et al. 2023). While these findings shed some light on the role of microtubules for the maintenance of stromulation, the first step, initiation of a new stromule at the outer envelope of the plastid body, has remained elusive, so far. We are currently investigating the role of *Nt*KP1, the tobacco homolog of KIS1 for stromule initiation and elongation.

### Do stromules channel oxylipin biosynthesis? Apparently, but not really

The compartmentalization of jasmonate biosynthesis across multiple organelles (Wasternack and Song 2017) creates a logistical challenge for plant cells, particularly during stress responses when rapid signal transduction is crucial. Stromules might facilitate this inter-organelle cross talk and potentially modulate MeJA-dependent gene expression by channeling the jasmonate biosynthetic pathway. Support for this hypothesis comes from our findings on subcellular localization: The transition of OPDA from the plastid into the peroxisome is prone to limit jasmonate biosynthesis, if OPDA would need to diffuse through the cytoplasm (according to Fick’s second law, the time required to cross a distance by free diffusion increases with the square of the distance). This bottleneck could be circumvented when peroxisomes attach to plastids, and when the plastidic exporter for OPDA is recruited to those sites, where the peroxisomes are found. In fact, we observed that JASSY, the bona fide OPDA exporter of plastids (Guan et al. 2019), was partitioned to stromules (Fig. 7). While the majority of stromules are contacting peroxisomes, only a subpopulation of peroxisomes is adjacent to stromules (Fig. 8a**–**c). However, this subset appears to be closely associated, as suggested by a NND analysis (Fig. 8d). Although this analysis supports nonrandom proximity, it does not by itself demonstrate persistent physical tethering; time-resolved contact analyses normalized to stromule length/membrane surface would be required to address this. These findings indicate that such close association would support the efficient transfer of OPDA to peroxisomes and might be sustained by the ER as suggested by the constriction of stromules at sites where they overlap with ER (Fig. 8F1–3). These constrictions are congruent with a model where stromules are guided through channels between ER tubules through membrane contact sites (Schattat et al. 2011).

The interaction of peroxisomes with chloroplasts, especially under light stress, is a well-known phenomenon (reviewed in [Bauwe et al. 2010]). A close interaction between these 2 organelles is also supported by the finding of specific dynamins shared for the division machinery of both chloroplasts and peroxisomes (Zhang and Hu, 2010). Furthermore, laser tweezer studies in leaves of *Arabidopsis* (Oikawa et al. 2015) and tobacco (Gao et al. 2016) demonstrated physical interaction. To what extent plastids link to peroxisomes in nonphotosynthetic cells, where photorespiration is not to be expected, remains unclear. A very recent study, investigating the dynamics of organelle interactions during genesis and degradation of glyoxysomes by high-quality electron microscopy (Gao et al. 2016), was able to unveil an intimate interaction of etioplasts with the ER, but did not come across interactions between glyoxysomes and plastids. There is very little information on the interaction between leucoplasts and peroxisomes. Actually, we were able to recover only one report (Barton et al. 2018), where a subpopulation of plastids in epidermal pavement cells of *Arabidopsis thaliana* was shown to be leucoplasts, evident from their lack of thylakoids. These leucoplasts did entertain interactions with both mitochondria and peroxisomes, and they also produced stromules. However, the authors did not find a preferential interaction of peroxisomes with stromules. To what extent these plastids that coexist with chloroplasts can be equalled to the leucoplasts in tobacco BY-2 cells that are completely void of any chlorophyll is difficult to tell, as well what their physiological context might be.

While the proximity of peroxisomes with stromules would favor a model of metabolic channeling, the data for gene expression show a more complex situation. When stromulation is suppressed by oryzalin, the induction of *AOC*, *JAZ1*, and *JAZ3* by MeJA is amplified (Fig. 5d). This is consistent with stromulation modulating plastid-to-nucleus signaling and thereby regulating hormone responsiveness, rather than stromules directly inducing transcription. Since MeJA is exogenous, any impact of jasmonate synthesis is unlikely to contribute; the observed phenomenon is not to be explained by changes in hormone *level*, but by changes in hormone *responsiveness*. Thus, rather than providing improved conversion of a metabolic precursor, OPDA, for jasmonate biosynthesis, stromules seem to modulate a signal that shapes the response of key genes in jasmonate biosynthesis and response. This shifts the focus on retrograde signaling from plastids to the nucleus.

### Are stromules tools for operational retrograde signaling?

Most proteins acting in the plastid are encoded in the nucleus and need to be imported into the plastid posttranslationally. As a consequence of this spatial separation, the need of the plastid for specific proteins has to be conveyed to the respective genes in the nucleus, a phenomenon known as retrograde signaling. The retrograde signaling coordinating the establishment of photosynthetically active thylakoids has been intensively studied from the early 1980s. However, this so-called biogenic signaling needs to be complemented by a so-called operational signaling (Pogson et al. 2008) that maintains plastid functionality subsequent to its biogenesis. While biogenic signaling has been investigated in detail, including the recovery of specific mutants (for a recent review see [Loudya et al. 2024]), operational signaling has not obtained the same degree of attention. However, there is accumulating evidence for a role of OPDA in operational signaling (a concise summary of this phenomenon is given in [Kopriva 2013]).

Previously thought to be just a precursor of jasmonate, OPDA was later recognized as signal of its own virtue. Alternatively, OPDA can bind to its receptor, cyclophilin 20 (Park et al. 2007), and stimulate cysteine accumulation, a bottleneck for synthesis of the antioxidant glutathione. Glutathione is thought to relay the signal from the plastid to the nucleus and to activate a specific group of basic region leucine zipper transcription factors, termed TGA factors (named after their target *cis*-motif, TGACG). As to activate their downstream targets, TGA factors need COI1, which is also a central component of the complex activating JA-Ile–responsive genes (Stotz et al. 2013). Interestingly, one of these TGA-responsive genes is *OPR*, the peroxisomal enzyme channeling OPDA towards jasmonate synthesis. In other words, JA-Ile/JAZ-dependent and TGA-dependent gene activation compete for COI1 as a shared component. As a result, retrograde signaling triggered by the interaction of OPDA with its receptor, cyclophilin 20, in the stroma, is predicted to downmodulate JAZ-dependent gene expression.

We used oryzalin to remove microtubules. Since microtubules are needed to sustain stromules, we also suppress stromulation that otherwise would result from the MeJA treatment. We find that the stromules induced by MeJA attenuate the induction of *AOC*, *JAZ1*, and *JAZ3*. This would be consistent with a working model, where stromules promote the transport of glutathione from the plastid to the nucleus, activating TGA-dependent gene expression. If stromulation is intercepted by oryzalin, this retrograde signaling would be disrupted, and this should increase the pool of COI1 available for JA-Ile–dependent gene activation, culminating in higher steady-state levels for MeJA-induced transcripts. While the effects of oryzalin on the positioning of peroxisomes are conceivable, peroxisomes seem to be primarily under control of actin (Mathur et al. 2002). In that paper, the role of microtubules was explicitly addressed and excluded. In MeJA-treated, oryzalin strongly modulated the transcript accumulation of *AOC*, *JAZ1*, and *JAZ3* (Fig. 5d), consistent with a role for stromules in modulating MeJA-dependent gene expression. Under SA treatment, the impact of oryzalin appears to be gene specific (Fig. 5e) rather than showing a uniform amplification or repression. Thus, the oryzalin effects on transcript levels are more consistent with differential modulation of hormone-responsive gene expression than with a nonspecific transcriptional effect (Fig. 5d–e). A similar role of stromules in communicating from plastids to the nucleus has been proposed for the hypersensitive reaction (for a recent review, see [Jung et al. 2024]). Such a communicative function might involve passage of specific metabolites through the stromule envelope. However, it might as well employ exchange of membrane materials with other organelles. Here, a closer analysis of dynamics by fluorescent recovery after photobleaching might give interesting insights.

The question, how a physical entity (stromules) can modulate gene expression, is crucial. We propose here that this modulation stems from spatial reorganization. Whether the retrograde signal channelled by stromules is linked with glutathione is currently investigated. Independently of the nature of this signal, stromulation can serve as a paradigm that architecture can shape signal transduction.

## Materials and methods

### Generation of marker lines for stromules and peroxisomes

To visualize stromules and peroxisomes, 4 strategies were employed: (i) Marker tpFNR-mEOS was based on an N-terminal fragment of ferredoxin NADPH oxidoreductase, including a transit peptide (Marques et al. 2003), fused to the photoconvertible mEOS protein (Jaideep Mathur et al. 2010; Schattat et al. 2012). (ii) The protein SFR2 fused to RFP (Moellering et al. 2010) labelled the outer plastid membrane (Mathur et al. 2022). Both constructs were kindly provided by Prof. Dr. Jaideep Mathur as well. (iii) To label peroxisomes and stromules simultaneously, we used a vector where the peroxisome targeting signal 1 (PTS1), fused with mCherry at its N-terminus, and the stroma marker described in (i), here fused to monomeric enhanced GFP (mEGFP) (Zacharias et al. 2002), were combined in tandem using a Golden Gate cloning strategy, kindly provided by Dr. Martin Hartmut Schattat (Halle University). (iv) A GFP fusion of the tobacco ortholog for the OPDA exporter JASSY (Guan et al. 2019) was used to visualize potential export sites at the outer envelope. This GFP fusion was constructed via Gateway from a full-length JASSY mRNA template (bp 265–1269 of XM_016595169.1) purified by the Universal RNA Kit (Roboklon), after reverse transcription with the M-MuLV cDNA Synthesis Kit (NEB). The target sequence was amplified using primers extended by the GATEWAY recombination sites. The primer sequences were:

5′-GGGGACAAGTTTGTACAAAAAAGCAGGCTTCATGAGAACGGAGGGTCAGAG-3′

and

5′-GGGGACCACTTTGTACAAGAAAGCTGGGTCCGATGACTTGGCAATCAAAGAC-3′

The amplicon could then be integrated into the Gateway pDONR vector by virtue of the recombination sites. After testing the transitions by sequencing, the insert could be transferred into the destination vector pK7FWG2 (Karimi et al. 2002) by the recombinase reaction enabled by the Gateway routine. This destination vector provided kanamycin resistance in plant cells, a fusion of GFP to the C-terminus of JASSY, and constitutive expression by the CaMV 35S promoter.

The 4 markers were transformed into tobacco BY-2 cells using an *Agrobacterium tumefaciens* strain LBA4404 as described by Klotz and Nick (2012) with minor modifications. After co-cultivation of BY-2 cells with *Agrobacterium*, transgenic cells were selected for 4 dadys in liquid MS medium containing 300 mg/mL cefotaxime sodium, and 50 mg/mL kanamycin (for JASSY-EGFP), along with 30 mg/mL hygromycin (for SFR2-mRFP, PTS1, and tpFNR-mEOS). The cells were then transferred to solid MS medium and further selected for 2 weeks in the dark on medium containing the respective antibiotics. Then, the calli were screened for the presence of the target gene by PCR and sequencing, before being cultivated in suspension and, as a backup, on plates, always in the presence of the respective antibiotics (Table 1).

**Table 1:** Sequences of the oligonucleotide primers used for RT-qPCR and GenBank accession numbers for the genes analyzed in the current study.

Gene target	mRNA accession no.	Forward primer 5ʹ–3ʹ	Reverse primer 5ʹ–-3ʹ
***Nt-JAZ1***	AB433896	CCAATTGCGAGACGAAATTCACTTAC	CCAAGCCATGCCTTATTTTCCTCATTC
***Nt-JAZ2***	AB433897	GCAGCACCTGCTCAACTGACC	GCACCACATTAGGAGGAACGCAACC
***Nt-JAZ3***	AB433898	GGATTCCGGTCGATTCGCCG	CCAAGGCTGAGATCTCCAAAGGAAC
***Nt-OPR3***	XM_016592560	GGTAGGGTTGGAAGTGAAGAGG	ATGACACAAGATCAGCATCACC
***Nt-AOC4***	XM_016586618	AGAGAATTGGAATAACAGCAGG	AAGTGTCCTCGTAAGTCAAGT
***Nt-L25***	L18908	GTTGCCAAGGCTGTCAAGTCAGG	GCACTAATACGAGGGTACTTGGGG

### Cell culture

Tobacco BY-2 cells (*Nicotiana tabacum* L. cv. “Bright Yellow 2') were cultured in a modified Murashige and Skoog medium with 4.3 g·L^−1^ Murashige and Skoog salts (Duchefa, Haarlem, the Netherlands) with 3% w/v sucrose as carbon source, supplemented with 200 g·L^−1^ KH_2_PO_4_, 100 m g·L^−1^ inositol, 1 m g·L^−1^ thiamine, and 0.9 µM 2,4-dichlorophenoxy acid as auxin. The pH was adjusted to 5.8. Cells were subcultivated weekly, inoculating ∼0.5-g fresh weight into 30 mL in 100-mL Erlenmeyer flasks. The cells grew at 26 °C in the dark under constant shaking on a KS260 basic orbital shaker (IKA Labortechnik, Staufen) at 150 rpm. In case of transgenic lines, the medium was complemented with the respective antibiotics. Every 3 wks, the backup BY-2 calli were subcultured on media solidified with 0.8% (w/v) agar (Roth, http://www.carlroth.com).

### Treatment with phytohormones and inhibitors

Methyl jasmonate (Sigma-Aldrich, Deisenhofen, Germany) was dissolved from a commercial stock of 4.2 M in 50% ethanol to final concentrations of 30 to 100 µM. The maximal concentration of the solvent was 0.05%. Salicylic acid (Sigma-Aldrich, Deisenhofen, Germany) was prepared as a 1 M stock solution in DMSO s 0.1%. Oryzalin (Chem Service, Inc., West Chester, Pennsylvania, United States), inhibiting microtubule polymerization, was used at 10 µM and diluted from a 10 mM stock in DMSO, a concentration that completely eliminates microtubules (Dembele et al. 2025). Latrunculin B (Sigma-Aldrich, Germany), inhibiting actin polymerization, was used at 2 µM, and diluted from a 1 mM stock in DMSO. Again, this concentration fully saturates actin elimination (Durst et al. 2014). Phenidone (Sigma-Aldrich, Germany), inhibiting jasmonate biosynthesis by blocking lipoxygenases, was used at 2 mM and dissolved from a 1 M stock in DMSO. Therefore, residual concentrations of DMSO were 0.1% for oryzalin, and 0.2% for latrunculin B and phenidone. Therefore, solvent controls were conducted with 0.2% DMSO. Cells were treated at d 4 after subculturing, ie, when the majority of cells had stopped cycling and were in G_1_, had initiated cell expansion. In case of phenidone, cells were treated for 30 min, for the other hormones and inhibitors; treatment time was 60 min. Cells were treated at d 4 after subcultivation, at the onset of the expansion phase, and remained on the orbital shaker at 150 rpm at 26 °C in the dark.

### Visualization of actin

Actin filaments were labelled after mild fixation as described in Maisch and Nick (2007). Briefly, 0.5 mL of suspended BY-2 cells were fixed for 10 min in 1.8% w/v paraformaldehyde prepared freshly in 0.1 M PIPES, with 5 mM MgCl_2_, and 10 mM EGTA, pH 7. Then cells were permeabilized with 1% glycerol in the same buffer for 10 min. After washing twice 10 min in the same buffer, the cells were incubated with 0.66 μM TexasRed-Phalloidin (Sigma-Aldrich Chemie GmbH, Munich, Germany) for 35 min in the dark. The unbound dye was washed out 3 times for 10 min in phosphate-buffered saline (0.15 M NaCl, 2.7 mM KCl, 1.2 mM KH_2_PO_4_, and 6.5 mM Na_2_HPO_4_, pH 7.2, adjusted with NaOH), and then the cells were mounted for examination under a spinning disc microscope. To see actin in the relation to stromules, BY-2 cells expressing tpFNR fused with mEOS as a stroma marker were preincubated with 100 µM MeJA for an hour before fixation and staining for actin filaments.

### RNA extraction and reverse transcription quantitative PCR analysis

Cells were treated with the different hormones and inhibitors as described above. To remove the supernatant, the cells were harvested by filtration through a Büchner funnel via short-time vacuum (10 s) under room temperature, and the cellular residue directly harvested into liquid nitrogen. The frozen cells were homogenized (TissueLyser), and total RNA was extracted using a commercial kit (Universal RNA Kit, Roboklon), adhering to the guidelines of the manufacturer. The extracted RNA was reversely transcribed into cDNA using the M-MuLV cDNA Synthesis Kit according to the protocol of the provider using 1 μg of total RNA as template. Steady-state transcript levels for target genes were determined by RT-qPCR as described in Hazman et al. (2015) in a Bio-Rad CFX Connect Real-Time PCR Cycler (Bio-Rad, Munich, Germany). Targets were the jasmonate response genes *JAZ1*, *JAZ2*, and *JAZ3* as proxy for the accumulation of JA-Ile. Additionally, *AOC4*, representing the last plastidic step, and *OPR3*, representing the first peroxisomal step of jasmonate biosynthesis, were investigated. To normalize expression levels, L25 coding for a ribosomal protein was used as a housekeeping gene. Details for the genes and oligonucleotide primers are given in Table 1. Steady-state transcript levels were quantified relatively by means of the 2^−ΔCt^ method (Livak and Schmittgen 2001). Results represent mean and standard error from 3 independent biological replicates, each in technical triplicate.

### Statistical analysis of transcript data

Differences between treatments were analyzed for statistical significance using multivariate ANOVA, followed by Duncan's multiple range test with a significance level set at *P* < 0.01 by means of the IBM SPSS Statistics 22 software, utilized for these statistical evaluations.

### Confocal live-cell imaging

Confocal z-stacks of representative BY-2 cells were collected with an AxioObserver Z1 inverted microscope (Zeiss, Jena, Germany) equipped with a spinning disc device (Yokogawa CSU-X1 Spinning Disc Unit, Yokogawa Electric Corporation, Tokyo, Japan) and a Plan-Apochromat 63x/1.40 Oil DIC M27 (oil objective). For the excitation of EGFP, the 488-nm line was used; for the excitation of mCherry or mRF12, the 590-nm lines of an Ar-Kr laser were used. The images were saved in CZI and TIFF formats for subsequent analysis. Processing involving cropping and adjustments to brightness/contrast using Adobe Photoshop.

### AI-assisted quantification of stromulation

To investigate stromulation on a quantitative base, an AI-assisted strategy was employed, making use of the Apeer/arivis Cloud system (Zeiss, Jena, Germany) based on a training set of 100 CZI images of cells with stromules. The system was trained for pixel-based semantic segmentation by Apeer/ZEISS arivis Cloud. Subsequently, employing Zeiss ZEN desk version 3.6 software, the trained model was validated using negative and positive controls. This AI-assisted quantification was then applied to measure stromulation in cells subjected to various hormone and inhibitor treatment, or to follow the stromulation responses over different stages of the cultivation cycle (details in Supplementary Methods). If not stated otherwise, individual data points represent samples of 30 individual cells. The frequency distribution for stromule length was strongly left-skewed, with very short values being overrepresented. Therefore, we used the nonparametric Fligner-Killeen (Fligner and Killeen 1976) test to probe for statistical significance of different treatments.

### Nearest neighbor distance analysis

The spatial relationship between peroxisomes and stromules was measured by calculating the shortest Euclidean distance from each peroxisome to the nearest stromule using ImageJ. To control for randomness, peroxisomes were repositioned within the same image boundaries, providing a basis for comparison. Cumulative distribution functions (CDFs) illustrated clustering tendencies. Statistical analysis was done in R (version 4.5.1; R Core Team, 2025), using 2-sample t-tests and Kolmogorov–Smirnov tests to distinguish real spatial patterns from random distributions.

### Accession numbers

Sequence data from this article can be found in the GenBank/EMBL data libraries under the accession numbers listed in Table 1 for *Nt-JAZ1*, *Nt-JAZ2*, *Nt-JAZ3*, *Nt-OPR3*, *Nt-AOC4*, and *Nt-L25*, and under XM016595169.1 for Nt-JASSY.

## Supplementary Material

kiag373_Supplementary_Data

## Data Availability

The data underlying this article are available from the corresponding author on reasonable request.
