# Supplementary material for: Fingers for signaling? A possible role of stromules in intracellular communication
Source: Plant Physiol. 2026 Jun 16;201(4):kiag373. doi: 10.1093/plphys/kiag373 (PMC13524073; doi:10.1093/plphys/kiag373)
Supplement: Supplementary file 1 [file kiag373_supplementary_data.pdf]

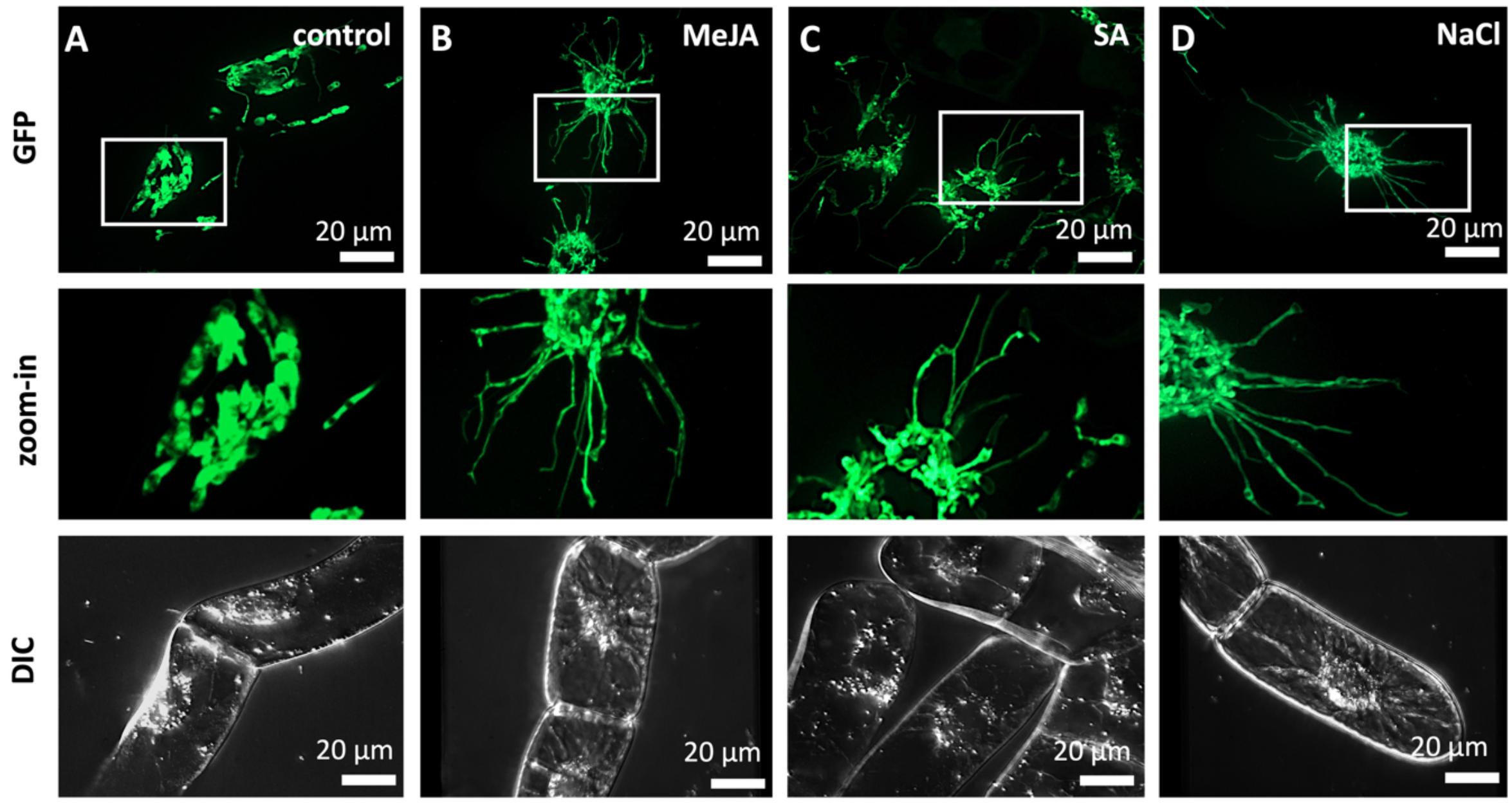

Supplementary Figure 1. Induction of stromulation by stress-related phytohormones and salinity in tobacco BY-2 cells. Geometric projections of confocal z-stacks for representative cells expressing the N-terminal fragment of ferredoxin NADPH oxidoreductase (tpFNR-mEOS) localized in the stroma in fusion with eGFP. **A** Untreated control cell. **B** Cell after treatment with 100 µM of Methyl Jasmonate (MeJA) for 1 h. **C** Cell after treatment with 100 µM of Salicylic Acid (SA) for 1 h. **D** Cell after treatment with 100 mM NaCl for 1 h. The zoom-in refers to the white square in the GFP image. A Differential Interference Contrast (DIC) image of the same cell is shown to connect the topology of the stromule signal.

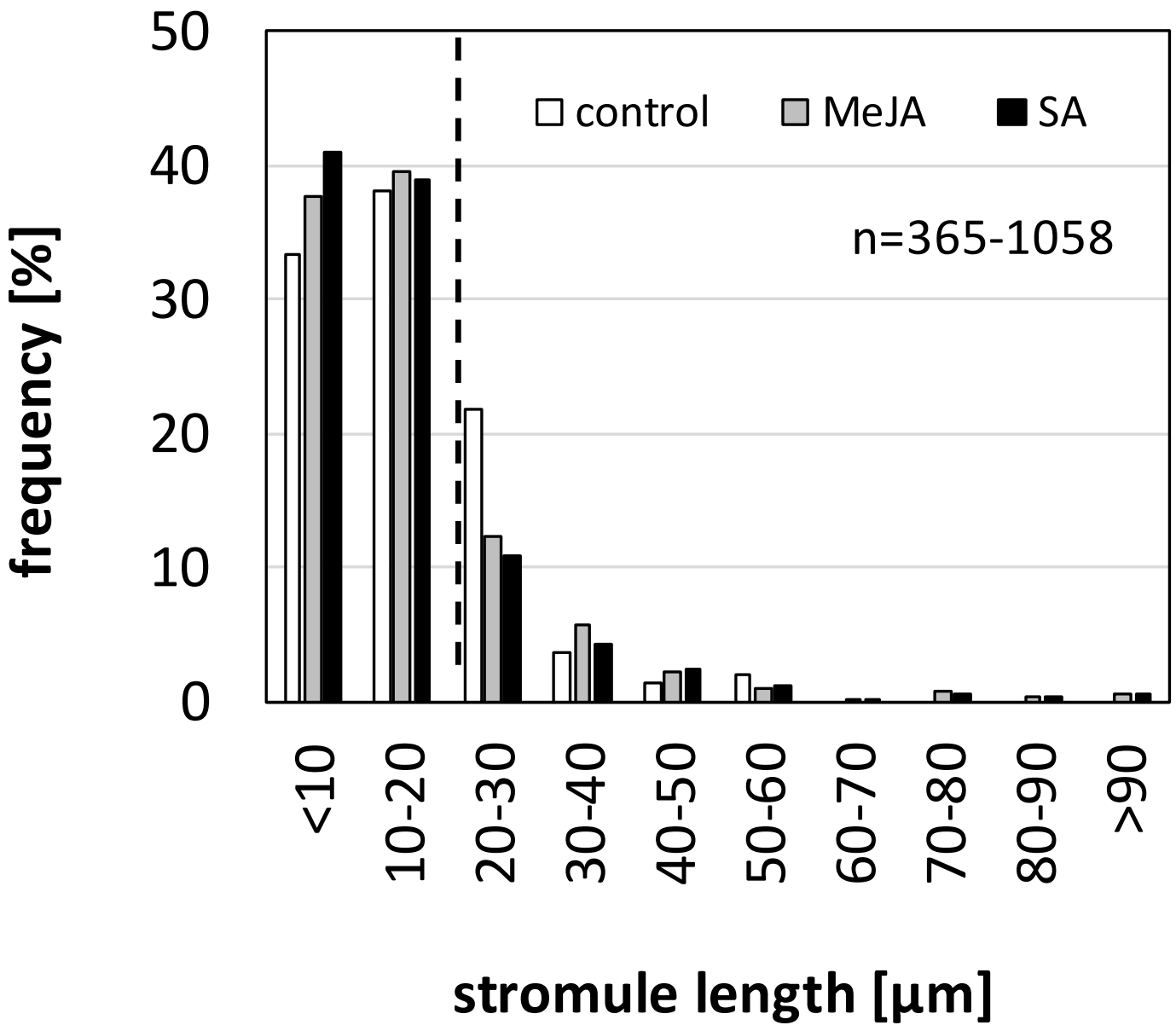

Supplementary Figure S2. Frequency distribution of stromule length in control cells (white bars), cells treated with 100 µM MeJA for 60 min (grey bars), or cells treated with 100 µM SA for 60 min (black bars). Dashed line: cut-off used for quantification due to the strong overrepresentation of very short stromules in all sample populations (representing between 365 and 1058 individual measurements). The difference in sample size is caused by the fact that the incidence of stromules differed depending on the treatment.

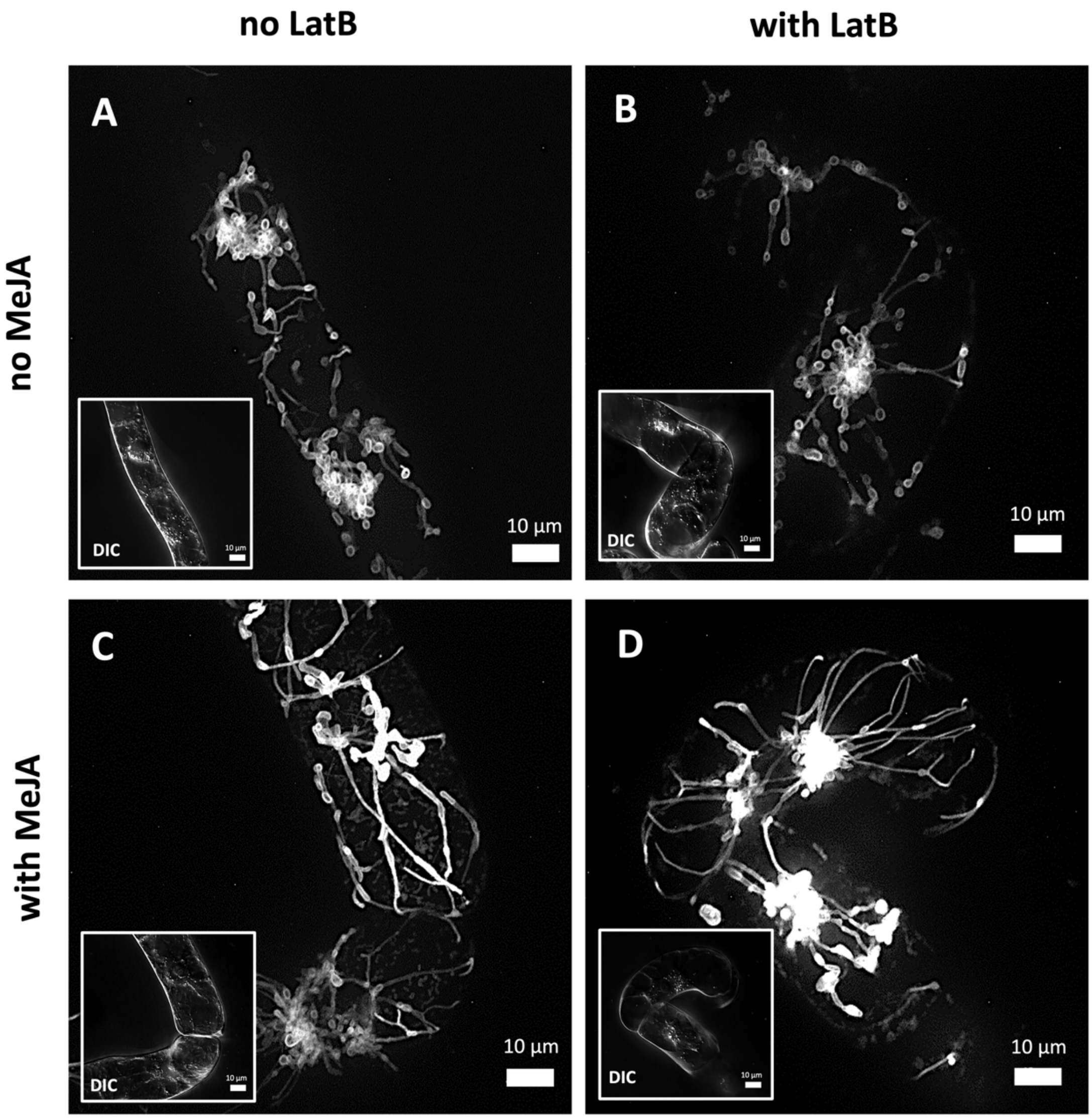

Supplementary Figure S3. MeJA-induced stromulation cannot be blocked by Latrunculin B, a compound eliminating actin filaments. Representative tobacco BY-2 cells expressing Sensitive to Freezing 2 (SFR2) localised in the outer plastid membrane in fusion with mRFP are shown. Images are geometric projections of confocal z-stacks. **A** Untreated control. **B** Treatment with 2 µM of Latrunculin B for 120 min. **C** Treatment with 100 µM of MeJA for 60 min. **D** Treatment with 100 µM of MeJA following a pre-treatment with 2 µM of Latrunculin B for 60 min. A Differential Interference Contrast (DIC) imaging of the same cell within each panel shows a topological comparison of stromule signal.

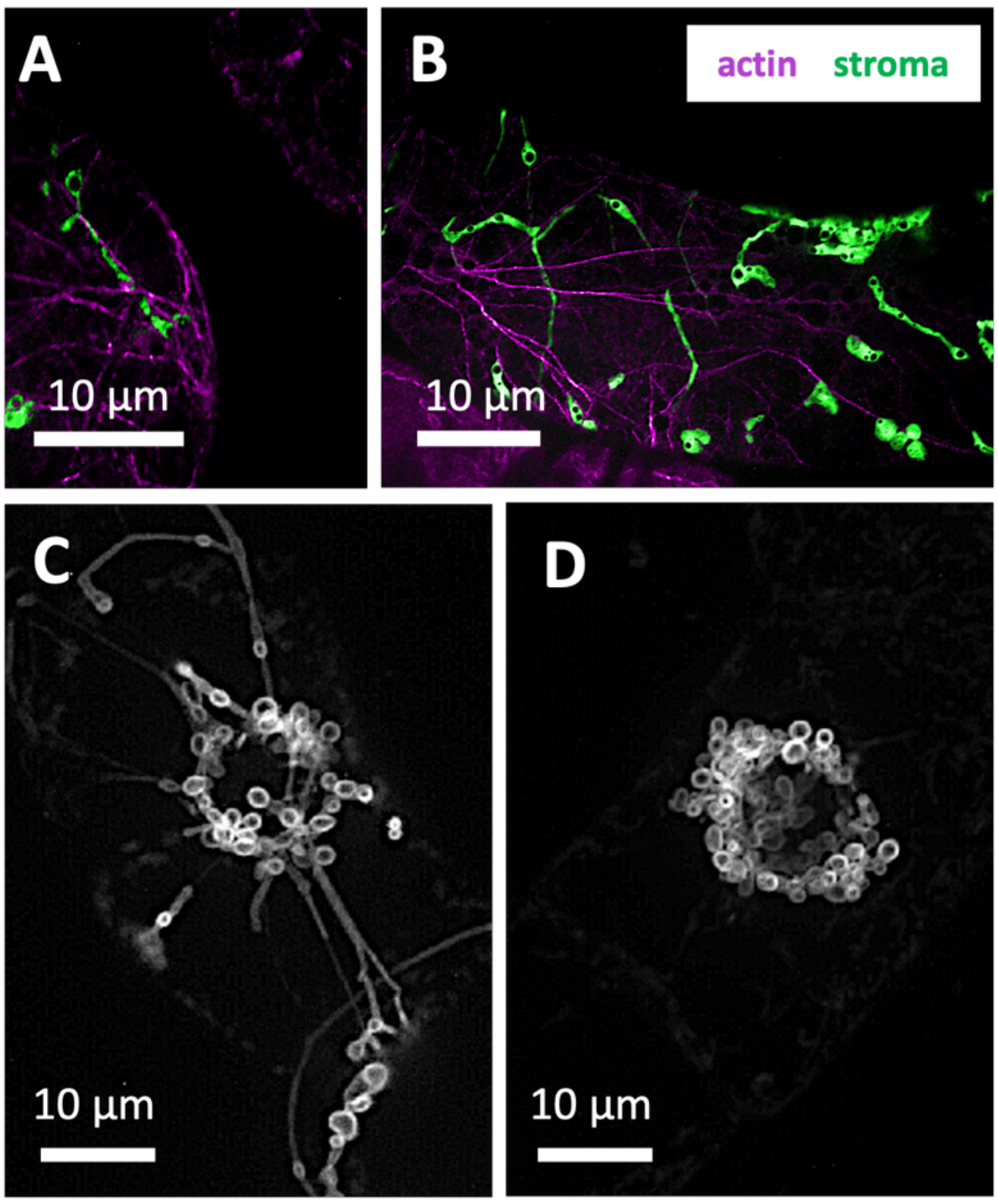

Supplementary Figure S4. Role of actin in stromulation induced by MeJA. **A, B** Double visualisation of stroma (tpFNR-mEOS, green) with actin filaments (TexasRed phalloidin, shown in magenta). **A** shows one of the rare cases, where stromules align with actin filaments, and **B** shows the more common situation, where they do not align. **C, D** Effect of cytoskeleton inhibitors Latrunculin B (**C**) and, for comparison, Oryzalin (**D**) on stromulation. The actin-filament inhibitor Latrunculin B (2 µM), and the microtubule inhibitor Oryzalin (10 µM) were added 60 min prior to stromule induction by MeJA (100 µM, 60 min).

Supplementary Methods

AI-assisted quantification of stromulation.

- **Training Strategy:** To quantify stromulation accurately, the AI model was trained on a carefully curated selection of 100 images sourced from BY-2 cells on the fourth day after subcultivation, marking the expansion phase when stromule responsiveness peaks. This dataset deliberately encompassed a broad range of morphological diversity within the expansion phase, allowing the model to capture authentic cell-to-cell variability while remaining sensitive to phase-specific features.
- **Semantic Segmentation Approach:** For identifying stromules, we adopted a pixel-based semantic segmentation workflow using the Apeer/arivis Cloud platform. Rather than relying on cell-cycle indicators, the AI distinguishes stromules by their defining morphological characteristics, specifically, slender tubular structures with diameters less than 1 µm displaying a prominent stroma signal. By focusing on these geometric criteria, the algorithm inherently corrects for phase-related cellular differences, enabling robust recognition of stromule geometry despite minor morphological variations among expansion-phase cells.
- **Validation Controls:** To verify reliability, validation was conducted using both negative and positive controls. Negative controls consisted of division-phase cells presenting minimal stromulation, while positive controls involved hormone-treated expansion-phase cells abundant in stromules. The model was only deemed suitable for quantitative analysis when it consistently and clearly differentiated between these conditions.
- **Experimental Design:** All comparative treatments, including MeJA, SA, and Oryzalin, were administered synchronously to day-4 cell cultures, minimizing confounding growth-phase effects and ensuring that any observed changes reflected true treatment responses. The resulting statistical analyses, which revealed treatment-induced differences exceeding 2.5-fold with P-values below 0.05, confirm that this AI-based quantification reliably detects stromule-specific responses distinct from developmental background variation.
